# Microglial cGAS Deletion Preserves Intercellular Communication and Alleviates Amyloid‐β‐Induced Pathogenesis of Alzheimer's Disease

**DOI:** 10.1002/advs.202410910

**Published:** 2025-02-05

**Authors:** Sijia He, Xin Li, Namrata Mittra, Anindita Bhattacharjee, Hu Wang, Shujie Song, Shangang Zhao, Feng Liu, Xianlin Han

**Affiliations:** ^1^ Barshop Institute for Longevity and Aging Studies University of Texas Health Science Center at San Antonio San Antonio TX 78229 USA; ^2^ Department of Cellular and Integrative Physiology University of Texas Health Science Center at San Antonio San Antonio TX 78229 USA; ^3^ Division of Endocrinology Department of Medicine University of Texas Health Science Center at San Antonio San Antonio TX 78229 USA; ^4^ Metabolic Syndrome Research Center The Second Xiangya Hospital of Central South University Changsha Hunan 410011 China; ^5^ Division of Diabetes Department of Medicine University of Texas Health Science Center at San Antonio San Antonio TX 78229 USA

**Keywords:** Alzheimer's disease, cGAS, innate immune, microglia

## Abstract

Innate immune activation plays a crucial role in the pathogenesis of Alzheimer's disease (AD) and related dementias (ADRD). The cytosolic DNA sensing pathway, involving cGAMP synthase (cGAS) and Stimulator of Interferon Genes (STING), has emerged as a key mediator of neurodegenerative diseases. However, the precise mechanisms through which cGAS activation influences AD progression remain poorly understood. In this study, we observed significant up‐regulation of cGAS‐STING signaling pathway in AD. Notably, this increase is primarily attributed to microglia, rather than non‐microglial cell types. Using an inducible, microglia‐specific cGAS knockout mouse model in the 5xFAD background, we demonstrated that deleting microglial cGAS at the onset of amyloid‐β (Aβ) pathology profoundly restricts plaque accumulation and protects mice from Aβ‐induced cognitive impairment. Mechanistically, our study revealed cGAS promotes plaque‐associated microglia accumulation and is essential for inflammasome activation. Moreover, we showed that restricting cGAS‐mediated innate immunity is crucial for preserving inter‐cellular communication in the brain and induces pleiotrophin, a neuroprotective factor. These findings offer novel insights into the specific roles of the innate immune system in AD employing a cell‐type‐specific approach. The conclusions provide a foundation for targeted interventions to modulate the microglial cGAS‐STING signaling pathway, offering promising therapeutic strategy for AD treatment.

## Graphical abstract

Innate immune activation plays a vital role in the development of Alzheimer's disease, yet the mechanisms remain unclear. This study identifies microglial cGAMP synthase (cGAS) as a central driver of amyloid‐β pathology and unveils a novel microglia‐myelin crosstalk mediated by pleiotrophin. These findings provide groundbreaking insights into microglia's role in neurodegeneration via their impact on cell–cell communication.

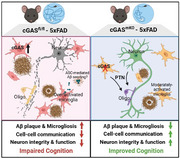

## 1 Introduction

Alzheimer's disease (AD), a devastating condition that impairs memory and cognitive function, is becoming increasingly prevalent among the elderly.^[^ ^1^ ^]^ Despite significant research efforts to understand the underlying causes of AD/ADRD (Alzheimer's disease and related dementias), no definitive cure has been discovered yet. Genome‐wide associated studies (GWASs) have identified a plethora of AD risk factors, which include triggering receptor‐expressed on myeloid cells 2 (Trem2), apolipoprotein E (APOE), ATP‐binding cassette transporter (ABCA) family, CD33, and complement pathway genes.^[^ ^2^ ^]^ Intriguingly, more than half of the risk loci that clearly involve a specific gene are significantly enriched or uniquely expressed in immune cells, particularly microglia and macrophages.^[^ ^3^ ^]^ This intriguing finding suggests that immune molecules play a pivotal role in the development and progression of the disease.^[^ ^3^ ^]^

The cGAS (encoded by *Mb21d1* gene)‐Stimulator of Interferon Genes (STING) pathway is an essential innate immune signaling for the initiation of type I interferon response against pathogen infections via sensing of double‐stranded DNA (dsDNA). Activated by the binding of self‐ or non‐self dsDNAs, cGAS catalyzes the production of 2′3’ cyclic GMP‐AMP (2′3’‐cGAMP), which in turn acts as a secondary messenger to activate STING. The activation of STING further promotes interferon gene expression by inducing the TBK1‐IRF3‐NFκB axis.^[^ ^4^ ^]^ In addition to its classical function in innate immunity against pathogen infection, the involvement of the cGAS‐STING pathway has been reported in various physiological and pathological processes such as autoimmunity,^[^ ^5^ ^]^ cellular senescence,^[^ ^6^ ^]^ autophagy,^[^ ^7^ ^]^ inflammation,^[^ ^8^ ^]^ and cancer immunity.^[^ ^9^ ^]^ Multiple studies have also emphasized its roles in neurological disorders, including multiple sclerosis,^[^ ^10^ ^]^ Parkinson's disease,^[^ ^11^ ^]^ amyotrophic lateral sclerosis,^[^ ^12^ ^]^ traumatic brain injury,^[^ ^13^ ^]^ stroke,^[^ ^14^ ^]^ and aging‐related neurodegeneration.^[^ ^15^ ^]^ Particularly, recent studies suggested a contribution of cGAS‐STING pathway activation in the development of AD,^[^ ^16^ ^]^ underscoring the significance of further understanding the roles of cGAS in AD pathogenesis. Given the essential involvement of innate immune pathways in AD, targeting to modulate the activity or levels of innate immune factors has been considered a promising strategy for preventing or delaying AD progression.^[^ ^17^ ^]^ Specifically, cGAS inhibitor TDI‐6570 has recently been demonstrated to improve memory function in an animal model of AD with tauopathy.^[^ ^16b^ ^]^ The significant potential of cGAS inhibitors as treatments in humans is further supported by its recent updates in therapeutic patent application,^[^ ^18^ ^]^ as well as the ongoing clinical trial.^[^ ^19^ ^]^ Meanwhile, this has also prompted great efforts in identifying new human cGAS inhibitors,^[^ ^20^ ^]^ allowing for further optimization based on desired pharmacological properties for their potential application in specific diseases. These progress, combined with basic scientific efforts in better understanding cGAS‐related disease mechanisms, greatly support the potential therapeutic value of targeting cGAS in the treatment of AD.

Despite being highly enriched in immune cells such as macrophages and microglia, multiple lines of evidence have demonstrated the diverse roles of cGAS in non‐immune cells. cGAS expression in central nervous system (CNS) neurons and peripheral nervous system (PNS) Schwann cells promotes spontaneous axon regeneration.^[^ ^21^ ^]^ Striatal neuronal cGAS‐STING signaling promotes inflammatory and autophagic responses in Huntington's disease.^[^ ^22^ ^]^ cGAS expression in human astrocytes mediates cellular response to foreign dsDNA.^[^ ^23^ ^]^ Activation of the cGAS‐STING pathway also plays a vital role in aging‐related endothelial dysfunction.^[^ ^24^ ^]^ These findings suggest a wide distribution of cGAS expression in the brain and the potential existence of cell type‐specific roles mediated by the cGAS‐STING pathway. Further, these cell type‐specific effects may act collectively in a diverse or synergistic manner in disease progression. Thus, identifying cell‐type specific functions is critical for understanding AD pathogenesis and the future development of targeted therapeutic strategy.

In the current study, we first identified that microglia are responsible for the augmentation of cGAS‐STING signaling in response to amyloid‐β (Aβ) pathology in the brain. By establishing an inducible, microglia‐specific *cGAS* knockout mouse model in the 5xFAD background, we investigated the effects of cGAS on Aβ pathology, as well as Aβ accumulation‐induced cognitive impairment. We further employed immunofluorescence (IF) staining to characterize the role of microglial cGAS in regulating microgliosis and the formation of dystrophic neurites in the context of Aβ pathology. Lastly, we elucidated the impact of microglial cGAS deficiency on the cell type composition as well as cell–cell communication of the brain at a single cell resolution, providing further mechanistic insights into how the innate immune pathway participates in the pathogenesis of AD. Findings from our study provide valuable information for understanding the novel role of microglial cGAS in modulating the pathogenesis of AD.

## 2 Results

### 2.1 Microglia are the Main Contributors to the Up‐regulated cGAS‐STING Pathway in AD Brain

**Figure 1:**
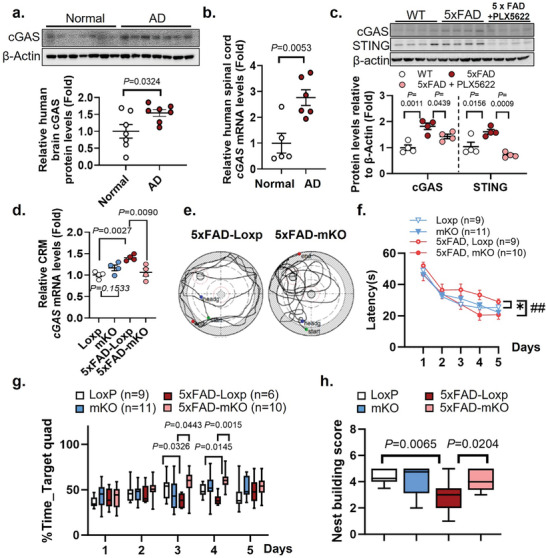
Microglia‐specific cGAS deletion protects mice from Aβ accumulation‐induced cognitive deficits. a) Western‐blot evaluation of cGAS protein levels in human postmortem brain samples. *n* = 7/group. b) qPCR measurement of *cGAS* mRNA levels from human spinal cord samples of normal and AD individuals. *n* = 5–6/group. c) Protein levels of cGAS and STING in mouse brain lysate. (Female, 9‐month‐old, *n* = 4/group). d) Cerebrum tissue mRNA levels of *cGAS* measured with qPCR. *n* = 4/genotype. e) Representative tracking, f) Latency (^##^ *P* = 0.0387, ^*^ *P* = 0.0409), and g) Percent time in the target quadrant during training of the MWM test. (Showing male data at 6 months old). h) Nest building test score. (7‐month‐old males and females, *n* = 8–12/group). Student's *t*‐test for (a,b), two‐way ANOVA followed with Šidák correction for (c,d,g, and h), repeated measures ANOVA for (f).

In our investigation of cGAS in AD, we initially assessed its expression in postmortem AD patient tissues and normal controls. As previously reported,^[^ ^16a^ ^]^ cGAS protein was elevated in the AD brain compared to controls (**Figure** **1**a). Consistent with this finding, we observed an increase of *cGAS* mRNA levels in AD spinal cord tissues (Figure 1b), a CNS region enriched with myelin. While cGAS expression has been traditionally associated with immune cells,^[^ ^4b^ ^]^ recent studies have suggested its involvement in non‐immune cells in the brain, such as neurons,^[^ ^21^ ^]^ astrocytes,^[^ ^23^ ^]^ and endothelial cells.^[^ ^24^ ^]^ Consistently, our analysis of human brain cortex data from online resources^[^ ^25^ ^]^ demonstrated high *cGAS* expression in microglia, moderate expression in neurons and oligodendrocytes, and detectable but low levels in astrocytes and endothelial cells (Figure S1a–c, Supporting Information). Similar expression patterns were observed in mouse spinal cord tissue based on data from a single cell sequencing study^[^ ^26^ ^]^ (Figure S1d, Supporting Information).

To determine the cell population responsible for cGAS elevation in AD, we treated 5xFAD mice with CSF1R inhibitor PLX5622, a compound known to selectively deplete microglia.^[^ ^27^ ^]^ In line with the induction of cGAS signaling observed in human AD, both cGAS and STING protein levels were significantly induced in 5xFAD mice compared to age‐matched wild‐type controls (Figure 1c). This induction was abolished upon microglial elimination in the PLX5622‐treated 5xFAD group (Figure 1c), suggesting that microglia are the primary contributors to the elevation of cGAS in AD brain.

### 2.2 Microglia‐Specific cGAS Deletion Protects Mice from Aβ Accumulation‐Induced Cognitive Deficits

To explore the potential impact of cGAS up‐regulation in the process of AD development, we generated a tamoxifen‐inducible, microglia‐specific cGAS knockout mouse line (mKO) by crossing Cx3cr1‐CreERT2 mice with cGAS flox/flox mice (Loxp) (Figure S2a, Supporting Information). The mKO was further crossed with 5xFAD mice to generate control and conditional knockout mice in an Aβ pathology background (denoted as 5xFAD‐Loxp and 5xFAD‐mKO, respectively). To avoid any developmental disruption, we injected mice with tamoxifen at 2 months of age to induce *Mb21d1* gene deletion in adult microglial populations. qPCR evaluation detected the induction of *cGAS* mRNA in 5xFAD‐Loxp versus Loxp, while this induction was abolished in the cerebrum (CRM) of 5xFAD‐mKO mice (Figure 1d), reflecting a successful establishment of the cGAS mKO model. Note that no significant difference in *cGAS* mRNA levels was detected between Loxp and mKO groups. This could be due to a relatively low cGAS expression under the non‐amyloidogenic condition. To characterize the effect of microglial cGAS deletion on cognitive function, we performed behavioral tests at 4 months post tamoxifen induction. Of special interest, mice lacking cGAS (5xFAD‐mKO) reached the hidden platform with significantly lower latency during the training phase than 5xFAD‐Loxp littermates in a Morris water maze (MWM) test (Figure 1e,f; Figure S2b, Supporting Information). This was also reflected by the increased time they spent in the target quadrant (Figure 1g; Figure S2c, Supporting Information). Due to a large variation, we did not notice any significant differences among groups during the probe test performed on day 6 (Figure S2d,e, Supporting Information). In addition, the deletion of microglial cGAS greatly improved performance in a nest building test (Figure 1h; Figure S2f, Supporting Information). Together, these results established a protective effect in the absence of microglial cGAS, suggesting that the induction of microglial cGAS in Aβ pathology is a strong driving factor for cognitive decline in AD.

### 2.3 Selective Microglial cGAS Ablation Significantly Decreases the Plaque Load in 5xFAD Mouse Brains

**Figure 2:**
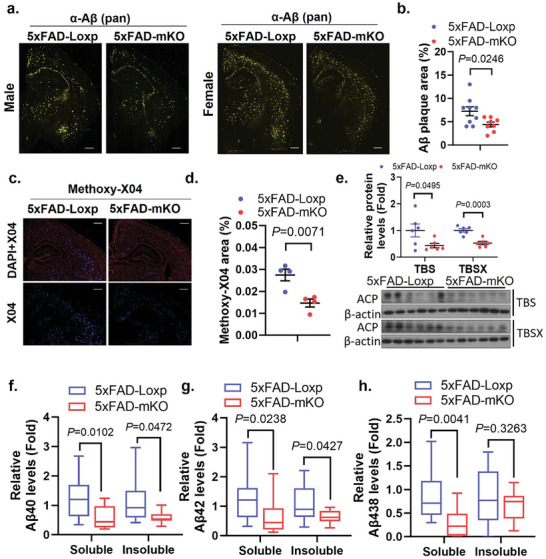
Selective microglial cGAS ablation significantly decreases plaque loads in 5xFAD mouse brains. a) Immunofluorescence staining of mouse cerebrum sections with a pan Aβ antibody to evaluate Aβ accumulation. Scale bars represent 500µm. b) Quantification of (a), *n* = 8–9/group, male and female combined, 7 months old. c) Evaluation of plaque loads using methoxy‐X04 staining (Male). Scale bars represent 500µm. d) Quantification of (c), *n* = 4/group. e) Western‐blot evaluation of APP cleavage product (ACP) levels in male cerebral TBS and TBSX lysates, *n* = 6/group. f) Aβ40, g) Aβ42, and h) Aβ38 levels in mouse brain lysates measured with ELISA. *n* = 11–12/group, male and female combined. A soluble fraction represents protein levels in TBS and TBSX combined equally, and insoluble fraction represents protein levels from GuHCl isolation. Student's *t*‐test for (b, d). Two‐way ANOVA followed with Šidák correction for (e–h).

In light of the improved behavior in 5xFAD‐mKO, we wondered if it was associated with altered Aβ pathology. By IF staining with an anti‐Aβ peptide antibody, we found a clear reduction of amyloid plaques overall in both male and female 5xFAD‐mKO versus 5xFAD‐Loxp littermates (**Figure** **2**a,b). A marked decrease of fibrillary Aβ in 5xFAD‐mKO brains was also observed by methoxy‐x04 staining (Figure 2c,d). Consistent with these findings, Western‐blot evaluation showed a significant decline of amyloid precursor protein (APP) cleavage products in the CRM lysate of 5xFAD‐mKO versus 5xFAD‐Loxp (Figure 2e). Additionally, both Aβ42 and Aβ40 levels were markedly decreased in soluble and insoluble fractions (Figure 2f,g), while Aβ38 was decreased in soluble fraction (Figure 2h), as determined by enzyme‐linked immunosorbent assay (ELISA). These observations suggest that microglial cGAS plays an important role in facilitating Aβ accumulation in AD pathology.

### 2.4 Conditional cGAS Deletion Blunts Microglial Responses to Aβ Pathology

**Figure 3:**
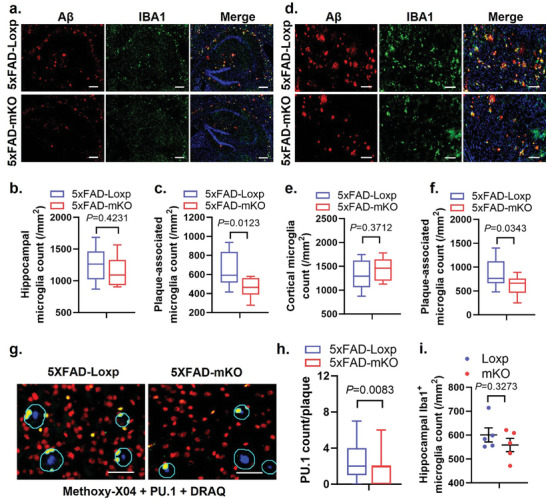
cGAS ablation ameliorates microgliosis in 5xFAD mouse brains. a) Hippocampal IF co‐staining using Aβ and IBA1 antibodies with DAPI (blue color) in 7‐month‐old mice. Scale bars represent 200µm. b) Quantification of overall hippocampal microglial density, and c) plaque‐associated microglial density in the hippocampus area, *n* = 10 mice/group. d) Cortical IF co‐staining using Aβ and IBA1 antibodies with DAPI. Scale bars represent 100µm. e) Quantification of overall cortical microglial density, and f) plaque‐associated microglia density in cortex area, *n* = 10 mice/group. g) Analysis of plaque‐associated microglia count within a 10 µm distance to plaques using methoxy‐X04 co‐staining with PU.1 and DRAQ (DNA stain indicating nuclei). Scale bars represent 50 µm. h) Quantification of g), *n* = 35 plaques/genotype. i) Quantification of IBA1 IF staining in Loxp and mKO mice without plaques, *n* = 5 mice/group. Student's *t*‐test for (b, c, e, f, h, and i).

We next asked whether cGAS reduction affects microglial activation in response to Aβ. IF co‐staining with IBA1, DAPI and Aβ antibodies revealed similar numbers of DAPI^+^ microglia in the hippocampal area of 5xFAD‐mKO and 5xFAD‐Loxp mice, while the numbers of microglia within a 10‐µm radius from plaque borders were significantly reduced in 5xFAD‐mKO compared to 5xFAD‐Loxp (**Figure** **3**a–c), suggesting decreased microglial reactivity in response to plaque deposition. A similar observation was also found in the cortex region of the brain (Figure 3d–f). The decline of plaque associated microglia was further evidenced by a decreased number of PU.1^+^ nuclei detected within a 10‐µm radius from methoxy‐x04^+^ plaque borders in 5xFAD‐mKO versus 5xFAD‐Loxp littermates (Figure 3g,h). Of note, no differences in microglial numbers or IBA1 staining were found when comparing mKO and Loxp mice without amyloid pathology (Figure 3i), suggesting that cGAS appears to be redundant for maintaining microglial numbers and activity in the steady state. These observations indicated that the presence of cGAS signaling is necessary for Aβ plaque‐induced microglial responses.

### 2.5 Loss of Microglial cGAS Attenuates Aβ‐induced Transcriptomic Signatures and Abolishes Microglial Inflammasome Activation

**Figure 4:**
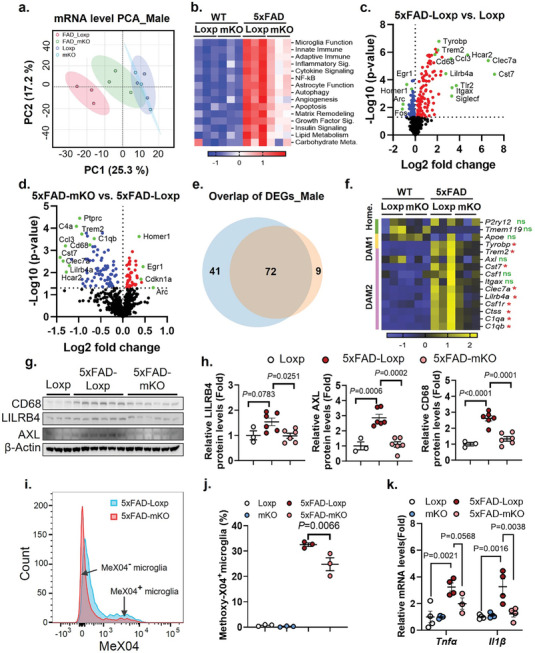
Loss of microglial cGAS limits Aβ‐induced transcriptomic signatures and suppresses microglial phagocytotic activity in 5xFAD mice. a) PCA analysis based on cerebrum tissue mRNA levels gathered from the NanoString neuroinflammation panel. (*n* = 3/group, 7‐month‐old males). b) Heatmap displaying pathway scores calculated based on cerebrum tissue NanoString data. c) Volcano plot showing DEGs by comparing 5xFAD‐Loxp to Loxp group. Red and blue color represents increased and decreased expression, respectively. The green color indicates genes of special interest. d) Volcano plot showing DEGs by comparing 5xFAD‐mKO to the 5xFAD‐Loxp group. e) Venn diagram showing overlap DEGs between plaque‐induced genes (5xFAD‐Loxp vs Loxp, blue color) and genes decreased upon cGAS deletion (5xFAD‐mKO vs 5xFAD‐Loxp, orange color) measured using NanoString (*p* ≤ 0.05). f) Microglial gene signature comparison among different genotypes based on NanoString counts. *ns* (not significant) or ^*^(*p* < 0.05) denotes the significance level by comparing 5xFAD‐mKO to 5xFAD‐Loxp group. g) Western‐blot evaluation of CD68, LILRB4, and AXL in 7‐month‐old male cerebrum tissues. h) Quantification of g) using β‐Actin levels as endogenous control. *n* = 3–6/group. i) Representative histogram of flow cytometry detection of microglia population with or without Aβ phagocytosis (Aβ marked by methoxy‐X04). j) Quantification of the proportion of Aβ phagocytotic microglia among genotypes (Male, 5‐month‐old, *n* = 3/genotype). k) qPCR detection of *TNFα* and *IL1β* expression levels in mouse cerebrum samples (*n* = 3–4/group, male, 7‐month‐old). One‐way ANOVA followed with Tukey's test for (h). Two‐way ANOVA followed with Šidák correction for (j) and (k).

Neuroinflammation is one of the fundamental features of AD. Activation of microglia in response to Aβ elicits expression of inflammatory and anti‐inflammatory cytokines, which play multifarious roles in neurodegeneration and neuroprotection.^[^ ^28^ ^]^ Given the reduced microglial response to plaques in the microglial cGAS‐deficient mice, we attempted to understand the mechanistic regulation of such a phenotype. We evaluated the gene expression profile of mouse cerebrum tissue using the NanoString neuroinflammation panel. Principle component analysis (PCA) showed a clear separation of the male 5xFAD‐Loxp group compared to groups without Aβ pathology (Loxp and mKO), while the loss of cGAS in a 5xFAD background led to an overlap of 95% confidence interval with Loxp controls (**Figure** **4**a). An overall pathway analysis indicated a drastically altered functional pathways between 5xFAD‐mKO and 5xFAD‐Loxp, the most significantly‐impacted pathways were related to immune and inflammation regulation (Figure 4b). We also detected marked changes in pathways including astrocyte function, autophagy, and lipid and carbohydrate metabolism (Figure 4b). Similar alterations between genotypes were also observed in females (Figure S3a,b, Supporting Information). Differential expressed gene (DEG) analysis identified 185 significantly changed genes (p ≤ 0.05) by comparing 5xFAD‐Loxp to Loxp, among which 113 were up‐regulated (including multiple microglial markers with highest fold changes, such as *Trem2, Tyrobp, Cd68, and Clec7a*), and 72 genes were down‐regulated (neuronal markers such as *Homer1* and *Fos* were found among the top decreased list) (Figure 4c). A separate DEG comparison of 5xFAD‐mKO versus 5xFAD‐Loxp showed a total of 123 genes significantly altered (p ≤ 0.05), among which 42 genes were up‐regulated (including *Homer1*), and 81 genes were down‐regulated (including microglial genes *Trem2, C1qb, Cd68, and Clec7a*) (Figure 4d). Note that the DEG list generated from 5xFAD‐Loxp versus Loxp comparison was highly overlapped with the DEG list generated by comparing 5xFAD‐mKO to 5xFAD‐Loxp (Figure S3c, Supporting Information). Moreover, among the 113 genes that were induced by Aβ pathology (5xFAD‐Loxp vs Loxp), 72 were down‐regulated by cGAS deletion (5xFAD‐mKO vs 5xFAD‐Loxp) (Figure 4e). A further query of these 72 genes in the WiKiPathway database indicated a strong association with microglial pathogen phagocytosis function (Figure S3d, Supporting Information).

Increased disease‐associated microglia (DAM) has been well‐established as a prominent feature in AD.^[^ ^29^ ^]^ Strikingly, we found that loss of microglial cGAS in the 5xFAD background abolished the induction of multiple Aβ pathology‐associated DAM markers, while the homeostatic microglial population appears to be intact (Figure 4f). Further protein level evaluation of DAM makers showed a decline of AXL, LILRB4, TREM2 and phagocytotic marker CD68 in 5xFAD‐mKO versus 5xFAD‐Loxp (Figure 4g,h; Figure S3e, Supporting Information). To further evaluate the impact of cGAS deletion on microglial function, we performed an in vivo phagocytosis assay. As shown in Figure 4i,j, loss of cGAS led to a significant decline of microglial phagocytotic activity toward Aβ. This suggests that cGAS is an important modulator for DAM formation and microglial phagocytotic function in Aβ pathology.

In addition to the uptake of cellular debris and Aβ via phagocytosis, microglia are also known to facilitate the propagation of Aβ pathology^[^ ^30^ ^]^ through mechanisms including activation of the NLRP3 inflammasome.^[^ ^31^ ^]^ We evaluated the levels of ASC (a.k.a PYCARD), an adaptor protein essential for NLRP3 inflammasome formation. IF staining showed that brain ASC levels were elevated in 5xFAD‐Loxp compared to the Loxp group, while cGAS deletion markedly decreased the levels of ASC (Figure S3f,g, Supporting Information). Consistently, cleaved‐Caspase 1 levels were also down‐regulated in 5xFAD‐mKO versus 5xFAD‐Loxp (Figure S3h,i, Supporting Information), suggesting lack of cGAS abolishes inflammasome activation in Aβ pathology. Consistently, qPCR analysis detected a marked down‐regulation of inflammatory gene expression (*TNFα, IL1β*), suggesting resolved neuroinflammation upon cGAS deletion (Figure 4k). Together, these results elucidated that cGAS is indispensable for inflammasome activation and neuroinflammation in Aβ pathology.

Similar to our findings in males, NanoString analysis in female CRM tissues identified a total of 174 genes that were induced by Aβ pathology (5xFAD‐Loxp vs Loxp), 56 of which were down‐regulated by cGAS knockout (5xFAD‐mKO vs 5xFAD) (Figure S4a–c, Supporting Information). These 56 genes were highly related to microglial phagocytosis and complement function according to WiKiPathway analysis (Figure S4d, Supporting Information). Further examination of the 25 genes that were consistently stimulated by Aβ and suppressed by cGAS deletion in both sexes (Figure S4e, Supporting Information) with the ChEA Transcription Factor Targets analysis revealed that the majority of these genes were downstream targets of the IRF8 transcription factor (Figure S4f, Supporting Information). As a microglia‐specific transcription factor in the CNS, IRF8 is critical for microglial activation and motility.^[^ ^32^ ^]^ These findings suggest that the cGAS deficiency‐induced decline in microglial response to plaques observed in 5xFAD‐mKO (compared to 5xFAD‐Loxp) may be driven by IRF8 down‐regulation.

### 2.6 Selective Microglial cGAS Ablation Prevents Neuronal Axonal Swelling in Aβ Pathology

**Figure 5:**
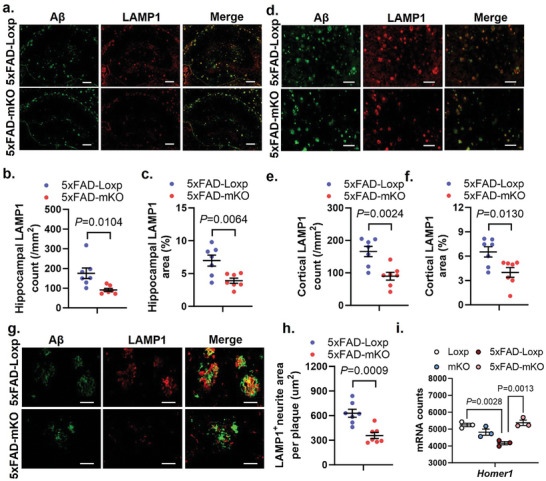
Selective microglial cGAS ablation prevents dystrophic neurites formation. a) Hippocampal IF co‐staining using Aβ and LAMP1 antibodies in 7‐months‐old mice. Scale bars represent 200µm. b) Quantification of overall hippocampal LAMP1 density, and c) percent area ratio in the hippocampus region, *n* = 7 mice/group. d) Cortical IF co‐staining using Aβ and LAMP1 antibody in 7‐months‐old mice. Scale bars represent 100µm. e) Quantification of overall cortical LAMP1 density, and f) percent area ratio in cortex region, *n* = 7 mice/group. g) IF staining, and h) Quantification of plaque‐associated LAMP1 levels, *n* = 7 mice/group. Scale bars represent 25µm. i) mRNA counts of *Homer1* gene measured with NanoString. *n* = 3/genotype. Student's *t*‐test for (b, c, e, f, and h). Two‐way ANOVA followed with Šidák correction for i).

Formation of dystrophic neurites is an important feature reflecting swollen neuronal axons^[^ ^33^ ^]^ that are often seen around plaques,^[^ ^34^ ^]^ which can be characterized by enhanced lysosomal marker LAMP1.^[^ ^35^ ^]^ Using IF staining, we detected a markedly decreased LAMP1 signal in the hippocampal area of 5xFAD‐mKO mice compared to that of 5xFAD‐Loxp (**Figure** **5**a–c). Similarly, the number of LAMP1^+^ areas and the ratio of the area to the respective total field of view significantly declined in the cortex of 5xFAD‐mKO mice (Figure 5d–f). Moreover, the amount of individual plaque associated‐LAMP1^+^ signal was greatly decreased in mice with microglial cGAS deficiency (Figure 5g,h). In parallel with the limited dystrophic neurites, the expression of *Homer1*, an important scaffolding molecule of the post synaptic density (PSD), was largely preserved in 5xFAD‐mKO mice brains versus 5xFAD‐Loxp (Figure 5i).

### 2.7 Cell–Cell Communication was Preserved via Microglial Innate Immunity Restriction in 5XFAD Brains as Indicated by Computational Prediction

To comprehensively survey the impact of cGAS deletion on brain cell profiles, we employed single‐nucleus RNA sequencing (snRNA‐seq) to decipher the composition and functions of major brain cell populations. From cerebrum tissues of 7‐month‐old Loxp, mKO, 5xFAD‐Loxp, and 5xFAD‐mKO mice, we collected a total of 62800 nuclei that passed quality control. As visualized in a Uniform Manifold Approximation and Projection (UMAP), unsupervised clustering followed by manual annotation revealed a total of 7 distinct major clusters across all samples (Figure S5a, Supporting Information). Each of the clusters was enriched with nuclei that highly express literature‐established cell‐type‐specific markers, such as *Sv2b* (Excitatory neurons, ExN), *Gad2* (Inhibitory neurons, InN), *Slc1a3* (Astrocytes, Astro), *Plp1* (Oligodendrocytes, Oligo), *Hexb* (Microglia, Micro), and *Pdgfra* (Oligodendrocyte progenitor cell, OPC) (Figure S5b, Supporting Information). An unbiased comparison among clusters generated a list of top expressed genes which further confirmed their cell type identity. For example, *Nhs* and *Satb2* are known markers of InN and ExN, while *Mobp* and *Inpp5d* are well‐established Oligo‐ and Micro‐ specific genes, respectively (Figure S5c, Supporting Information). Consistent with previous snRNA‐seq datasets,^[^ ^36^ ^]^ we detected a relatively high ratio of neurons (above 80% of total nuclei) compared to other glial cell types in all samples (Figure S5d, Supporting Information).

**Figure 6:**
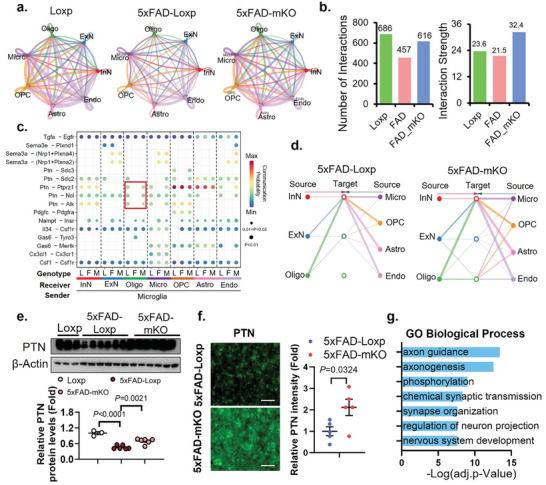
Deletion of microglial cGAS preserves Aβ accumulation‐induced loss of cell–cell communication in 5xFAD brain. a) Circle plot displaying the number of interactions among all major cell types from each genotype group. The thickness of lines represents the relative quantity of intracellular communication. b) Inferred total number of interactions and interaction strength in each group. c) Bubble plot showing the probability of ligand‐receptor interaction derived from microglia to each of the other 6 cell types (For the genotype label, L: Loxp; F: 5xFAD‐Loxp; M: 5xFAD‐mKO). d) Hierarchy plot of PTN signaling among cells in different genetic groups. e) Western‐blot evaluation and quantification of PTN protein levels in cerebrum tissues (male, 7‐month‐old, *n* = 3–6/genotype). f) IF evaluation and quantification of PTN protein levels and distribution in brain sections (7‐month‐old, *n* = 5/genotype). Scale bars represent 50µm. g) GO biological process analysis of DEGs (5xFAD‐mKO vs 5xFAD‐Loxp) from oligodendrocyte population. One‐way ANOVA followed with Tukey's test for (e). Student's *t*‐test for (f). Differential expression analysis g) was generated using model‐based analysis of single‐cell transcriptomics (MAST) test in the Seurat package.

To understand the mechanism(s) by which microglial cGAS deletion impacts major cell types in the brain, we investigated intercellular communication using CellChat.^[^ ^37^ ^]^ By referencing the secreted signaling database which contains 2019 mouse ligand‐receptor pairs, we found multiple autocrine and paracrine activities among the 7 major cell types in all genotypes (**Figure** **6**a). Quantified analysis showed a predicted overall decline of the number and strength of interactions in 5xFAD‐Loxp compared to Loxp, which was completely recovered by the deletion of microglial cGAS in the 5xFAD‐mKO mice (Figure 6b; Figure S6a,b, Supporting Information). Pathways that were consistently impacted in both human and mouse AD, recovered to normal levels in the 5xFAD‐mKO group, encompassing bone morphogenetic protein (BMP) signaling, fibroblast growth factor (FGF) signaling, pleiotrophin (PTN) signaling, neuregulin (NRG) signaling, and growth arrest‐specific (GAS) signaling (Figure S6c,d, Supporting Information).

To explore how microglia signals to other cell types, we generated a bubble plot designating microglia as the source cell and all other cell types as the target cells (Figure 6c). Among the significant ligand‐receptor communication pairs, we observed several interaction patterns: 1) the Cx3cl1 signaling, known to be specific for microglia, was only detected in the microglia population, validating the robustness of our analysis; 2) the Sema3‐Nrp1 and Nampt‐Insr signalings, absent only in the Loxp group, suggesting a strong correlation with Aβ pathology; and 3) the PTN signaling, exclusively absent in the 5xFAD‐Loxp group, indicating a strong negative association with cognitive deterioration (Figure 6c). Notably, the change in the PTN signaling appeared to be most significant toward oligodendrocytes, which is further illustrated by a hierarchy plot demonstrating the re‐establishment of the PTN signaling targeting oligodendrocytes in 5xFAD‐mKO compared to 5xFAD‐Loxp (Figure 6d). In support of this observation, a marked elevation of PTN protein level was detected by western‐blot analysis in the CRM of 5xFAD‐mKO versus 5xFAD‐Loxp mice (Figure 6e). Consistently, IF staining showed a significant induction of PTN in brain sections of mice lacking microglial cGAS (Figure 6f). PTN is known as a neurotrophic factor^[^ ^38^ ^]^ which also functions to promote myelin repairment.^[^ ^39^ ^]^ Based on this, the induction of PTN signaling in 5xFAD‐mKO mice may facilitate maintaining myelin and neuronal integrity. In line with this hypothesis, DEG analysis of the oligodendrocyte population (5xFAD‐mKO vs 5xFAD‐Loxp) showed that cGAS deletion significantly altered genes involved in axon guidance, synapse organization and neuron projection (Figure 6g). Future investigation using PTN depletion/inhibition in the 5xFAD‐mKO model may help to demonstrate the contribution of PTN elevation to the improved phenotypes observed in 5xFAD‐mKO mice.

To achieve a more comprehensive view of the consequences of altered cell–cell communication upon the loss of cGAS, we generated a scatter plot illustrating the incoming and outgoing interaction strength of each cell type in all genotypes (Figure S7a, Supporting Information). By analyzing the sum interaction strength (Figure S7b, Supporting Information) and comparing between genotypes, we identified oligodendrocytes as the most active cell type in terms of secretory signaling communications, followed by astrocytes, microglia, and endothelial cells (Figure S7c, Supporting Information). A signaling pattern heatmap was generated to examine the alterations in multiple secretory signaling pathways among different groups (Figure S7d, Supporting Information). From this analysis, we observed that the top altered pathway in oligodendrocytes was FGF signaling. With a detailed circle plot of FGF signaling shown in Figure S7e (Supporting Information), we found that FGF secretory pathway originated from oligodendrocytes were markedly enhanced in disease condition (5xFAD‐Loxp vs Loxp) and returned to base level in the absence of cGAS (5xFAD‐mKO vs 5xFAD‐Loxp). On the contrary, other pathways such as GAS‐MerTK signaling, which is known to suppress neuroinflammation,^[^ ^33^ ^]^ were greatly diminished in amyloid pathology and were re‐established with cGAS deletion (Figure S7f, Supporting Information). These observations demonstrated that the dynamic cell–cell communication network disrupted by disease pathology can be largely restored by the deletion of microglial cGAS.

### 2.8 Knockout of Microglial cGAS Protects Against Neuronal Loss in 5XFAD Mice

**Figure 7:**
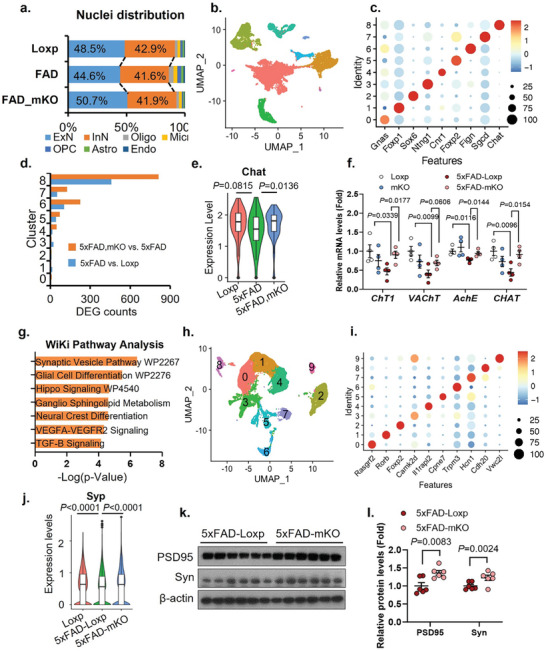
Suppression of microglial innate immunity protects the integrity of neuronal populations in mice. a) Bar graph showing the frequency of each cell type in each genotype. b) UMAP showing sub‐clusters of inhibitory neuron population. c) Dot plot evaluating the expression of established sub type‐specific marker genes of inhibitory neurons. d) Bar graph showing DEG counts generated by indicated comparison pairs. e) Violin plot comparing the expression level of the *Chat* gene in cluster 8 among different genotypes. f) qPCR analysis of cholinergic neuron markers expression in cerebrum lysates (*n* = 6/genotype). g) WiKi pathway analysis using shared gene lists that are oppositely regulated in the following two comparisons: 5xFAD‐Loxp versus Loxp, and 5xFAD‐mKO versus 5xFAD‐Loxp. h) UMAP showing sub‐clusters from the excitatory neuron population. i) Dot plot displaying the expression of sub type‐specific marker genes from excitatory neurons. j) *Syp* expression in excitatory neuron population. k) Western‐blot of PSD95 and synaptophysin protein levels from mouse brain lysates, *n* = 6/genotype. l) Quantification of (k). Mouse semi‐cerebrum samples (*n* = 3) were pooled for sequencing of each genotype for snRNA‐seq. Two‐way ANOVA followed with Šidák correction for (f) and (l). *p*‐values for differential expression analysis between different clusters in (e) and (j) were calculated using the Mann‐Whitney U test.

Mice lacking microglial cGAS exhibited improved spatial memory learning function (Figure 1e–g), suggesting a preserved neuronal network under Aβ pathology. The preserved cell–cell communication and elevated PTN signaling in 5xFAD‐mKO mice prompted us to further explore the impact of cGAS deletion on neuronal integrity. By comparing different groups from our snRNA‐seq data, we detected a notable decrease in the frequencies of ExN and InN nuclei in the presence of amyloid pathology (5xFAD‐Loxp vs Loxp) (**Figure** **7**a). Meanwhile, a near complete recovery was observed in the absence of cGAS (5xFAD‐mKO vs 5xFAD‐Loxp) (Figure 7a). This finding aligns with data from the Religious Order Study (ROS) cohort (ROSMAP),^[^ ^25c^ ^]^ revealing a decline in the frequency of neuronal cell populations in AD compared to normal brains (Figure S8a, Supporting Information). These results demonstrated that neuron loss is a critical common feature in human AD and mouse amyloid pathology. Moreover, deletion of cGAS specifically in microglia is sufficient to rescue the decline of neurons under pathological conditions.

In addition to examining alterations in neuronal numbers, we also investigated whether the deletion of microglial cGAS modulates the transcriptome profile of neurons. Using unsupervised clustering, we identified nine distinctive inhibitory neuron sub‐clusters (Figure 7b), with each representing a previously characterized inhibitory neuron sub‐type^[^ ^40^ ^]^ (Figure 7c). By analyzing DEGs on a per‐sub‐cluster basis, specifically comparing 5xFAD‐Loxp versus Loxp (reflecting Aβ effect) and 5xFAD‐mKO versus 5xFAD‐Loxp (reflecting cGAS dependence), we observed that a *Chat* ^+^ sub‐cluster exhibited the highest number of DEGs among all groups (Figure 7d; Figure S8b, Supporting Information). This suggests that the status of these cells was most significantly influenced by both Aβ pathology and cGAS deletion. *Chat* ^+^ neurons actually mark a group of cholinergic neurons that produce acetylcholine (ACh). Severe loss of cholinergic neurons has been consistently observed in AD and has been proposed to contribute to memory and attention deficits.^[^ ^41^ ^]^ To this end, we detected a clear reduction of *Chat* expression in amyloid pathology, while cGAS deletion preserved *Chat* levels (Figure 7e). Consistently, using the qPCR method, we detected a significant loss of cholinergic neuronal markers in the CRM homogenate of 5xFAD‐Loxp mice compared to Loxp littermates, including choline acetyltransferase (*ChAT*), vesicular acetylcholine transporter (*VAChT*), acetylcholinesterase (*AChE*), and choline transporter (*CHT1*), while deletion of microglial cGAS almost completely rescued the decline of these cholinergic gene expression (Figure 7f). To further understand how cellular function was impacted, we performed a query of the 122 cGAS dependent *Chat* ^+^ neuron DEGs (including 64 induced in 5xFAD and 58 suppressed in 5xFAD) that were altered by Aβ pathology (Figure S8c,d, Supporting Information) in WiKiPathway and KEGG databases. We found that these genes were largely involved in pathways associated with synaptic vesicle, glycosphingolipid biosynthesis, and axon guidance (Figure 7g; Figure S8e, Supporting Information). These findings indicate that the impairment of cholinergic neurons in AD depends on cGAS. By inhibiting microglial cGAS, it is possible to protect inhibitory neuron function, potentially by preserving acetylcholine production and maintaining the synaptic vesicle recycling system.

In the excitatory neuron population, we have identified a total of 10 sub‐clusters expressing specific markers (Figure 7h,i). Meanwhile, we found that the levels of presynaptic marker *Synaptophysin* were down‐regulated in 5xFAD‐Loxp compared to Loxp, while significantly elevated in 5xFAD‐mKO nuclei compared to that of 5xFAD‐Loxp (Figure 7j). The neuroprotective effect of cGAS ablation was also evidenced by the elevated PSD95 and Synaptophysin protein levels from brain lysates of 5xFAD‐mKO mice (Figure 7k,l). Together, these findings indicate that restricting microglial cGAS is essential for preserving neuronal number and function in Aβ pathology.

## 3 Discussion

The involvement of the cGAS‐STING pathway in Aβ and tau pathologies has recently been realized via studies performed in a mouse model of cGAS whole‐body knockout.^[^ ^16^ ^]^ Yet a critical knowledge gap exists on how cGAS participate in AD progression. With the goal of providing further mechanistic insights, we are the first group sought to understand the function of cGAS in specific brain cell types by employing an inducible, microglia‐specific cGAS knockout model. Our study emphasized the pivotal roles of microglial cGAS in the development of AD. Conditional deletion of cGAS in microglia limited Aβ deposition, significantly alleviated neuroinflammation, preserved intracellular communication, and reduced neuronal damage, which led to improved cognitive behavioral outcomes in mice. Through cell–cell communication studies, we have also identified a novel microglia‐oligodendrocyte crosstalk that participates in disease progression. We believe findings from the current study provide novel insights into the understanding of the role of microglia during AD pathogenesis, and offers new options for the therapeutic targeting of cGAS‐STING pathway in AD.

The Aβ peptide deposition‐induced formation of extracellular senile plaques is a hallmark of AD.^[^ ^42^ ^]^ Accumulating evidence from genetic, biochemical, and animal model studies has strongly suggested that excessive Aβ accumulation plays a central role in AD pathogenesis.^[^ ^43^ ^]^ In this study, we demonstrated that the absence of cGAS in microglia leads to a reduction in Aβ plaque load, suggesting that cGAS is involved in regulating the balance between Aβ accumulation and clearance. Multiple lines of evidence suggest that microglia play complicated roles in Aβ pathology: Microglia are the major cell type that mediates Aβ clearance through phagocytosis activities;^[^ ^44^ ^]^ conversely, microglia can facilitate propagation of Aβ pathology via seeding;^[^ ^30^, ^31^ ^]^ studies have also reported that microglia produce a substantial amount of APP, which can be cleaved internally to produce Aβ.^[^ ^45^ ^]^ We detected an overall decreased reactivity and in vivo phagocytotic activity in 5xFAD‐mKO compared to 5xFAD‐Loxp, indicating that up‐take of Aβ may not be the major mechanism for the decline of plaque load in 5xFAD‐mKO mice. Meanwhile, loss of cGAS substantially diminished inflammasome activity (reflected by the decrease of cleaved‐Caspase1 levels), further study with inflammasome inhibition is necessary to determine if cGAS deletion restricts the propagation of Aβ pathology via limiting inflammasome activation‐mediated Aβ seeding.^[^ ^31^ ^]^ On a parallel note, it was reported that the absence of microglia reduces the intensity of parenchymal Aβ plaques,^[^ ^46^ ^]^ while modulation of microglia‐enriched AD risk genes, such as knockout of Trem2, has shown inconsistent outcomes on amyloid or tau pathologies.^[^ ^47^ ^]^ For instance, Trem2 depletion^[^ ^48^ ^]^ or haplodeficiency^[^ ^47c^ ^]^ has been found to reduce early‐stage plaque compaction with limited impact on long‐term plaque load. A separate study showed that Trem2 deficiency reduces amyloid pathology in the early stage, but exacerbates it in the later stages of disease progression.^[^ ^49^ ^]^ On the contrary, one study using a Trem2 loss‐of‐function model demonstrated enhanced Aβ seeding associated with decreased phagocytotic activity during an early stage.^[^ ^47b^ ^]^ Among tau pathology related investigations, knockout of Trem2 in the PS19 human tau transgenic mouse has been shown to decrease microgliosis and limit brain atrophy without affecting phosphorylated tau or insoluble tau levels.^[^ ^47d^ ^]^ Trem2 deficiency in a nonmutant human tau background has been shown to exacerbate tau pathology through widespread dysregulation of neuronal stress kinase pathways.^[^ ^50^ ^]^ These inconsistencies suggest that disease stage, genetic background, and intervention method are crucial factors to consider when investigating the mechanisms of action of a microglial therapeutic target gene. Similar to the aforementioned knockout studies, the Trem2 elevation also has different effects at different stages of AD progression. For instance, induction of Trem2 suppresses amyloid accumulation and neuritic dystrophy at the pre‐plaque formation stage in 5xFAD mouse model (0–2 months), but it has no impact during plaque accumulating middle to late stages (2–6 months).^[^ ^51^ ^]^ This suggests that the plaque lowering effect of cGAS deletion at the plaque accumulating stage (2–6 months) in our study may be Trem2 independent. Notably, overexpressing microglial Trem2 at the pre‐plaque stage^[^ ^51^ ^]^ and suppressing microglial cGAS at plaque accumulating stages (this study) have both achieved beneficial effects of reducing amyloid deposition and neuritic dystrophy. Moreover, a common feature of the microglial profile shared by these beneficial modulations was the suppressed DAM signature.^[^ ^51^ ^]^ This suggests that restricting microglial activity at a moderate level throughout different disease stages maybe an ideal strategy to counteract Aβ‐accumulation and related pathology. Future efforts to enhance Trem2 activity during an early disease stage with combined therapy to suppress cGAS at the middle to late stages of the disease may help to maximize the therapeutic effects against AD.

Another important note is that, in addition to functioning as the downstream of cGAS, STING can also be activated in a cGAS‐independent manner.^[^ ^52^ ^]^ Whether cGAS‐independent STING activation plays an essential role in AD remains elusive. Nevertheless, while pharmacological inhibition of cGAS protects cognitive deficits in mice with tauopathy,^[^ ^16b^ ^]^ STING inhibition has also been shown to ameliorate AD symptoms in mice with amyloid pathology.^[^ ^16^, ^53^ ^]^ This further emphasizes the beneficial effects of restricting cGAS/STING during AD progression. It is also important to note that the benefits of limiting cGAS/STING have been investigated largely under specific pathogen‐free animal facilities. Given that cGAS is an important mediator of type I interferon response, whether cGAS inhibition has any innate immune‐related adverse effects in normal environments will require further study.

Our NanoString study revealed that microglia lacking cGAS exhibited a shifted gene expression profile that forms an intermediate state between homeostatic‐ and DAM‐like states. This is characterized by the preserved elevation of *Apoe*, *Csf1*, and *Itgax*, along with the abolished induction of DAM signatures including *Trem2*, *Clec7a*, *Csf1r*, AXL and LILRB4. Moderately activated microglia have been recognized for their neuroprotective effects in the early stages of AD by phagocytosis of Aβ deposits. However, this beneficial function declines upon excessive Aβ accumulation.^[^ ^54^ ^]^ In the late stages of AD, overactivated microglia contributes to elevated proinflammatory cytokine production and have reduced Aβ clearance capacity.^[^ ^55^ ^]^ Moreover, cell death of Aβ‐containing microglia contributes to plaque growth in AD.^[^ ^56^ ^]^ Given the decreased plaque load and inflammatory cytokine levels observed in 5xFAD‐mKO mice, it is reasonable to hypothesize that the loss of cGAS leads to a state of moderate microglial activation, which prevents the detrimental consequences of microglial overactivation. Further investigation focusing on a more detailed microglia‐specific, temporal transcriptomic analysis and functional study is warranted to test this hypothesis. Given that microglial activation is often concomitantly observed with the presence of astrogliosis and myelin disruption in AD, an additional consideration is whether there could be potential involvement of other glial cell types beyond microglia for the phenotype observed in this study. A recent study using a mouse model with complete microglia depletion concluded a limited impact on astrocytes with changes detected in an oligodendrocyte precursor cell population.^[^ ^46^, ^57^ ^]^ This appears to be in line with our findings from the intra‐cellular communication analysis discussed below.

A surprising observation from our cell–cell communication analysis was that oligodendrocytes are the most active cell type in response to microglial cGAS deletion. Indeed, they are the most versatile cells among both normal and disease conditions in terms of secretory signaling. As one of the major glia cell types in the brain, oligodendrocytes actively engage in cell–cell communications via ligand‐receptor signaling, which are essential for the maintenance of myelin, neuronal function, and overall brain homeostasis. Increasing lines of evidence have established myelin impairment as a prominent feature in AD,^[^ ^58^ ^]^ alongside tau neurofibrillary tangles, Aβ plaque, and neuroinflammation.^[^ ^59^ ^]^ A main feature of myelin sheath is its high lipid content. Changes in the myelin lipid profile without the presence of demyelination have been observed in the early stages of AD^[^ ^58a^ ^]^ and have been proposed to be an important contributor to disease initiation.^[^ ^58c^ ^]^ Moreover, these alterations have been found to enhance cytokines and secretory factors that contribute to neuroinflammation.^[^ ^58c^ ^]^ In line with this, our findings strongly suggest an intricate interaction among innate immunity, myelin integrity, and AD progression. We detected a complete loss of oligodendrocyte‐oriented PTN signaling in 5xFAD mice, which was re‐established in 5xFAD‐mKO mice. The PTN signaling pathway plays an important role in the survival, growth and differentiation of oligodendrytic lineage in the brain.^[^ ^60^ ^]^ Although further PTN depletion study is needed to clarify its involvement in the neuroprotective effect observed in 5xFAD‐mKO, the significant elevation of PTN implies that enhanced myelin integrity could contribute to the neuroprotective phenotype induced by limiting cGAS‐mediated microglial activation.

Neurodegeneration in AD can be characterized in twofold: increased neuronal death and altered neuronal function.^[^ ^61^ ^]^ snRNA‐seq analysis of datasets from current and previous studies revealed a consistent loss of neuron population in 5xFAD and human AD brains compared to respective controls. This aligns with the observation of loss of inter‐cellular communication in disease conditions, which was mainly contributed by the decline of both PTN and NRG, two top abundant secretory signaling pathways promoting neuron survival and neurogenesis.^[^ ^60^, ^62^ ^]^ Deletion of cGAS from microglia rescued the decline of PTN and NRG pathways, potentially explaining the recovery of neuronal content in the 5xFAD‐mKO mouse brain. In addition to the drastic changes in neuronal numbers, we also observed a robust protective effect on synaptic integrity in the brain of 5xFAD‐mKO mice compared to 5xFAD‐Loxp, as demonstrated by the preserved levels of *Homer1*, PSD95, and Synaptophysin upon cGAS deletion. This is in line with the observation of decreased plaque‐associated dystrophic neurites, which reflects the extent of damage to neuronal processes under Aβ‐induced neurotoxicity. These alterations are important indicators of improved neuroprotection, thus may be responsible for the improved performance of 5xFAD‐mKO mice in cognitive tests.

Additionally, microglia are known to interact with synapses under both physiological and pathological conditions.^[^ ^63^ ^]^ Removal of excessive synapses by microglia contributes to neuronal maturation during development.^[^ ^64^ ^]^ Meanwhile, microglial synaptic pruning has also been demonstrated to mediate synaptic loss in AD.^[^ ^65^ ^]^ The complement system plays a critical part in orchestrating this process through the labeling of synapses with C1q and its downstream molecule C3, followed by phagocytic elimination by microglia expressing complement 3 receptor (C3R).^[^ ^66^ ^]^ Notably, multiple complement pathway components, including *C1q*, *C4a*, and *C3ar1*, were found to decrease in 5xFAD‐mKO brain versus 5xFAD‐Loxp, supporting the notion that elevated microglial cGAS drives synaptic loss through up‐regulation of complement‐mediated synaptic pruning in AD. Thus, further investigation into the glia‐neuron interaction under cGAS‐deficient conditions may elucidate the detailed mechanisms behind the neuroprotective effects of targeting the cGAS‐STING pathway in AD.

## 4 Conclusion

In summary, our study has shed new light on the functional and mechanistic roles of cGAS in microglia‐mediated Aβ accumulation, neuroinflammation, disrupted cell–cell communication, and Aβ pathology‐induced synaptic damage (Figure S9, Supporting Information). Given the prevalence of AD and its vast impact on human health, our work offers a unique and innovative approach to interrogate the molecular basis of AD pathogenesis. This study could thus hold promise in identifying potential new drug‐targeting strategies and could pave the way for new avenues for AD treatment.

## 5 Experimental Section

### Sex as a Biological Variable

Our study examined male and female animals, and similar findings were reported for both sexes by comparing 5xFAD‐mKO versus 5xFAD‐Loxp groups within each sex. Note that higher plaque load and cognitive impairment have been detected in female 5xFAD compared to male 5xFAD, which was consistent with previous studies reporting female 5xFAD generally display more severe phenotype than males.^[^ ^67^ ^]^

### Human Samples and Study Approval

Postmortem human brain and spinal cord samples were provided by the NIH NeuroBioBank and Biggs Institute Brain Bank of UTHealth SA. For postmortem tissue collection, authorization from the donor or next‐of‐kin was collected to ensure that the wishes of the family and the deceased were respected. Use of postmortem human samples without individual identification was deemed exempt non‐human subject research by both NIH NeuroBioBank and UTHealth SA Institutional Review Board based on the human research protection regulation (45 CFR 46).

### Animal Model

All animal studies were performed in accordance with the guideline approved by the Institutional Animal Care and Use Committee (IACUC) of the University of Texas Health San Antonio (UTHSA), Protocol Number 20180044AP. Mice were provided with free access to food and water housed under 12/12 h light/dark cycles. Teklad laboratory diet (ENVIGO, Cat. #7012) was used to maintain mouse lines. The cGAS fl/fl mouse was obtained through collaboration. Briefly, it was generated by flanking partial exons of the *Mb21d1* gene with LoxP sequences. To generate the microglia‐specific cGAS knockout mouse, cGAS fl/fl mice were crossed with Cx3cr1‐CreERT2 mice (Stock No: 020940, the Jackson Laboratory, Bar Harbor, ME, USA). The resulted in cGAS fl/fl; Cx3cr1+/− mice were further crossed with 5xFAD (Stock No: 006554, the Jackson Laboratory) to generate 4 genotypes that were used in the current study: Loxp (cGAS fl/fl; Cx3cr1−/−), mKO (cGAS fl/fl; Cx3cr1+/−), 5xFAD‐Loxp (5xFAD; cGAS fl/fl; Cx3cr1−/−), and 5xFAD‐mKO (5xFAD; cGAS fl/fl; Cx3cr1+/−). All groups were given intraperitoneal injections of tamoxifen (80 mg kg^−1^ bodyweight) for 4 consecutive days at 2 months old to induce the knockout of the *Mb21d1* gene post developmental stage. All animal experiment groups were randomly assigned mice of the desired genotype.

### Nest Building Test

The nest building evaluation was performed according to the procedure described in the previous report^[^ ^68^ ^]^ with a 5‐point rating scale system. Mice were transferred to individual housing cage 1 h before the dark phase with free access to food and water. An intact piece of cotton sponge (3 g in weight) was provided to each mouse. Rating was performed the next morning based on the following criteria: 1) the amount and weight of intact sponge (pieces over 0.1 g); 2) the identification of nest site; and 3) nest wall circumference. When the criteria do not agree, a “split of difference” was granted by adding 0.5 in between scores.

### Morris Water Maze Test

The maze was set up using a water tank of 120 cm in diameter, in which a circular target platform (11 cm in diameter) was placed within the north‐west quadrant. Water was pre‐filled to 1 cm above the target platform surface one day before the experiment with white‐colored paint added to the water. Visual cues were placed around the tank at a height visible from the water level. Mice were acclimated in the behavior room for 1 h daily before trials. For training (spatial acquisition) days, four start positions (including N, S, E, and W) were semi‐randomized every day. Mice were placed in the water to explore for 1 min for each trial and were guided to the platform for a 15‐second‐stay if they did not find the platform within 1 min. Four trials were performed each day per mouse. Probe test was performed 24 h post the last training day, the target platform was removed, and each mouse was placed from the south position to complete a 1 min trial in order to assess spatial reference memory. All animals used in this test were free of visible health issues or injuries. The vision of mice was confirmed by 1 min swim with a visible cue on the platform after the MWM probe test, no mice had impaired vision. The speed of the swim was monitored as an indication of motor function, no mice were excluded based on this criterion. Another exclusion criterion was the lack of motivation to escape the water. Based on this, one female mouse was excluded from the test due to continuous floating in the water tank.

### mRNA Extraction and Gene Expression Analysis

Snap frozen cerebrum samples were cryofractured in liquid nitrogen. ≈10 mg of tissue per sample was used for extracting mRNA with TRIzol Reagent according to the manufacturer's instruction (Thermo Fisher Scientific, Cat. #15596018). RNA concentration was determined using a Spectrophotometer (DeNovix). RNA Integrity (RIN) was determined using the 4150 TapeStation system (Agilent) via RNA ScreenTapes. Subsequent gene expression using NanoString nCounter Technology was performed with Neuroinflammation Panels on nCounter SPRINT Profiler per manufacturer's instructions (NanoString Technologies). Initial data analysis was performed using nSolver Analysis Software 4.0, which was composed of background thresholding at the mean value of Negative Controls, Positive Control Normalization, and CodeSet Content Normalization. Additional analyses including Fold Change analysis between groups and pathway analysis were performed using nSolver Advanced Analysis Software 2.0. For qPCR measurement, 1 µg mRNA per sample was used to perform reverse transcription (Qiagen, Cat. # 205311). Gene expression levels were then detected using the SYBR Green (Applied Biosystems, Cat. #A25742) method using primer sequences listed in Table S1 (Supporting Information). Target gene levels were normalized to endogenous housekeeping gene *Gapdh* levels using the ΔΔCT method. Data were presented as fold change over respective controls.

### Immunoblotting Analysis

Frozen samples were cryofractured in liquid nitrogen, and 30 mg of tissue per sample was homogenized in N‐PER Neuronal Protein Extraction Reagent (Thermo Fisher Scientific, Cat. # 87792) with protease and phosphatase inhibitors (Thermo Fisher Scientific, Cat. # 78442). Samples were incubated on ice for 10 min and centrifuged at 12 000 x g for 15 min at 4 °C. Supernatant was collected for protein quantification using the BCA method. 30 µg protein/sample was resolved on NuPAGE 4–12% Bis‐Tris gels on the NuPAGE electrophoresis system (Life technologies). Proteins were then transferred onto PVDF membranes, blocked in 1% bovine serum albumin, and incubated with primary antibodies at 4 °C overnight. After washed with TBST, the blots were incubated with horseradish peroxidase‐conjugated secondary antibody and developed using the chemiluminescence (ECL) method (Thermo Fisher Scientific, Cat. # 32106). A list of antibodies used was included in Table S2 (Supporting Information).

### Immunofluorescence Staining

Brain tissues were collected upon harvest of animal post saline perfusion, incubated overnight in paraformaldehyde (4% v v^−1^), and dehydrated in 10, 20, and 30% sucrose buffer before imbedded into frozen blocks. Serial cross sections (10 µm) of the frozen tissue were collected followed by re‐hydration and citric acid‐based antigen retrieval. Sections were rehydrated and blocked with 10% goat serum (Sigma, Cat. # G9023) in PBST (PBS containing 0.05% Triton X‐100) at room temperature, incubated with a primary antibody with 5% goat serum‐PBST at 4 °C overnight in a humidified chamber. After being washed three times in PBST, slides were incubated with fluorescence‐labeled secondary antibody for 1 h at room temperature, before being mounted with ProLong Diamond Antifade Mountant with DAPI (Thermo Fisher Scientific, Cat. # P36971). Images were collected using a fluorescence microscope (KEYENCE, Cat. # BZ‐X800).

### Amyloid Beta Level Evaluation

Snap‐frozen whole cerebrum tissue was cryofractured in liquid nitrogen. Tissue (25–30 mg) was used for protein extraction according to the protocol described previously.^[^ ^69^ ^]^ Briefly, tissue was homogenized in 15 volumes of Tris‐buffered saline (TBS) and centrifuged at 1 00 000 × g for 1 h at 4 °C using a TLA‐55 rotor in an Optima TLX Ultracentrifuge (Beckman Coulter). The first supernatant, TBS‐soluble fraction, was frozen in liquid nitrogen and stored at −80 °C. Pellets were washed with 200 µL TBS buffer before re‐suspended in 15 volumes (w v^−1^ of tissue) of TBSX (TBS buffer containing 1% Triton X‐100) and mixed gently by rotation at 4 °C for 30 min. After being centrifuged at 1 00 000 × g for 1 h at 4 °C, the supernatant was collected as a TBSX‐soluble fraction. Pellets were re‐suspended into 400 µL of 5 M GuHCl, mixed by rotation at room temperature for 6 h, and span at 16 000 × g for 30 min, supernatant was collected as GuHCl‐soluble fraction. All buffers used were supplied with protease and phosphatase inhibitors (Calbiochem). Levels of Aβ peptides (amyloid‐β1–38, amyloid‐β1–40, amyloid‐β1–42) in each fraction were measured using V‐PLEX Aβ Peptide Panel 1 kit (Meso Scale Discovery, Cat. # K15200E‐1) according to manufacturer's instruction.

### In Vivo Aβ Phagocytosis

Mice were injected with methoxy‐X04 intraperitoneally at a dosage of 10 mg kg^−1^ bodyweight. 3 h post injection, mice were euthanized with isoflurane and perfused transcardially with 30 mL of ice‐cold PBS. Cerebrum tissue was dissociated by pre‐warmed enzymatic solution (containing 5U mL^−1^ Papain, 35U mL^−1^ DNaseI, and 1.65 mM L‐Cysteine in artificial cerebrospinal fluid (aCSF)) for a total of 40 min in a 37 °C water bath. The cell pellet was collected after centrifuge at 800 × g, 4 °C for 5 min, resuspended into 30% Percoll solution, and layered with 2 mL aCSF before centrifuged at 800 × g, 4 °C for 15 min to remove myelin and debris. Cell pellets were re‐suspended in 1xHBSS buffer, stained for dead cell (Invitrogen, Cat. # L10119), and labeled for microglia using CD11b‐APC (1:200) and CD45‐FITC (1:200) for 30 min at 4 °C. Cells were washed and suspended with 1xHBSS, flow cytometry was used to identity live microglial population (high in CD11b, low in CD45) based on respective single staining for compensation, and further evaluate the fluorescence signal of methoxy‐X04.

### Single‐Nucleus RNA Sequencing

Frozen mouse semi‐cerebrum tissues were used for nuclei isolation using a gradient centrifugation‐based method. Nuclei were pooled from 3 mice per genotype for each snRNAseq sample, and the PIPseq T20 3′ Single Cell RNA Kit v4.0 was used for cDNA library construction based on a particle‐templated emulsification method^[^ ^70^ ^]^ following manufacturer's instructions. Briefly, 40 000 nuclei per sample were gently mixed with barcoded hydrogel (PIPs), 1 mL partitioning oil was then added into the mixture before vortexed using a custom adaptor at 3000 rpm for 15 s horizontally and 2 min vertically. After removing excessive oil, the emulsion went through enzymatic lysis at 66 °C for 35 min and was broken by adding 750 µL breaking buffer. mRNA was then isolated with a de‐partitioning reagent, washed three times, and adjusted into 250 µL volume. For cDNA synthesis, reverse transcript (RT) mix containing template switch oligo (TSO) was added to the mRNA, incubated for 30 min at 20 °C and 90 min at 42 °C, followed by 10 min at 85 °C and a 4 °C hold.

Whole‐transcriptome amplification (WTA) was performed on purified reverse transcription product by adding WTA master mix and WTA primer thermocycling at (95 °C for 3 min, 16 cycles of 98 °C for 15 s, 69 °C for 4 min and 20 s, followed by 72 °C for 5 min and a hold at 4 °C). After WTA, barcoded hydrogel beads were removed using filter columns (6 min at 13 000 g), and amplified cDNA was purified using 0.8× SPRI, fragment analysis was performed on TapeStation to confirm the size range ≈1000bp. Libraries were generated with 500 ng cDNA using fragmentation (30 °C for 8 min followed with 65 °C for 30 min and a hold at 4 °C), adaptor ligation (20 °C for 15 min), and sample index PCR (98 °C for 45 s, 6 cycles of 98 °C for 15 s, 67 °C for 30 s, 69 °C for 45 s, followed by 72 °C for 1 min and hold at 4 °C). The product was purified using double sided SPRI purification (0.8× and 0.6×) to yield a final library with a fraction size ≈400 bp. Libraries were pooled and sequenced using an Illumina NovaSeq 6000 instrument with 1% PhiX using an S4 flow cell (150 pair‐end module).

### snRNA‐seq Data Analysis

Analysis of sequencing data was performed using PIPseeker software (v3.0.5) to generate gene expression matrices starting from processed FASTQ sequences, which include barcode identification, reference mapping, cell calling, and gene expression matrix generation. The gene expression matrices were then imported into R for downstream analysis using the Seurat package (v4.1.4).^[^ ^71^ ^]^ For quality control, nuclei with more than 5% mitochondrial gene content were removed. Nuclei with very low transcript counts were removed using a cut off of 1000 UMI and 500 genes. Genes that were detected in less than 10 nuclei were deleted. Potential doublets were identified and excluded using DoubletFinder.^[^ ^72^ ^]^ After filtering, a total nuclei count of 62 800 remained across 4 groups with a median of 11 610 UMIs and 3649 genes per nucleus. Data were then normalized (scale factor 10 000), log‐transformed, and scaled. PCA was performed using the top 2000 variable features, and the top 20 PCA components were used for UMAP analysis. Clustering was performed using the FindClusters function using resolutions ranging from 0.01 to 2. An ideal resolution was selected by marker gene evaluation with consideration from ClusterTree analysis. For identifying clusters, the expression levels of literature described cell type‐specific enriched marker genes in each cluster (*Plp1*, *Mbp*, *Cldn11*, *Mog* for Oligo; *Slc1a2*, *Gja1*, *Aqp4* for Astro; *Hexb*, *Csf1r*, *C1qa*, *P2ry12* for Micro; *Pdgfra*, *Vcan*, *Olig1* for OPC; *Flt1*, *Vtn*, *Cldn5* for Endo; *Grin1*, *Camk2a* for ExN; *Gad1*, *Pde10a*, *Sst*, *Npy* for InN) were evaluated and further confirmed with the list of top marker genes generated by FindMarkers function for consistency. For sub‐cluster annotation, marker genes from each sub‐cluster were submitted for query through enrichr databases and cross‐referenced with established sub‐set markers from literature. DEG analysis between conditions was performed using the MAST algorithm,^[^ ^73^ ^]^ which considers cellular detection rate as a source of nuisance variation.

### Cell–Cell Communication Analysis with Cell Chat

Cell–cell communication was evaluated using the CellChat tool^[^ ^37^ ^]^ (http://www.cellchat.org), which quantifies the propensity of cells to behave as a sender or receiver for numerous cell–cell signaling pathways. Ligand and receptor gene expression from our dataset was identified by query to an established secretory signaling CellChat database, interaction probabilities of cells were then calculated using law of mass model, communication networks can be presented using weighted directed graphs. The “computeCommunProb” function was utilized to calculate the interaction probability and displayed using the “netVisual” function. Using non‐negative matrix factorization (NMF), the numbers of communication patterns of each individual sample were speculated, extracted the major signaling inputs and outputs of all cell types, and visualized their distribution using the “netAnalysis_signalingRole_scatter” function among samples. For specific interest in certain cell types (for example, microglia), the “netVisual_bubble” function was employed, and set microglia as source cells to display ligand‐receptor pairs that have significant communication probability.

### Statistics

All data were shown as mean ± standard error of the mean (SEM) unless specified. For animal experiments, age‐ and sex‐matched mice were assigned to different treatment groups randomly to prevent potential bias. Data represent results from both male and female unless specified in figure legends. All biochemistry results were representative of at least 3 repeated experiments or as indicated. NanoString PCA analysis was performed using MetaboAnalyst 5.0. Statistical analysis was performed using GraphPad Prism 10. An unpaired two‐tailed *t*‐test was used for the comparison between two groups, for which *P* ≤ 0.05 was considered statistically significant.

One‐way ANOVA followed with Tukey's test, or two‐way ANOVA followed with Šidák correction was used to compare multiple groups, for which adjusted *p*‐values were displayed (labeled as *P* in the plots), *P* ≤ 0.05 was considered statistically significant.

## Conflict of Interest

The authors declare no conflict of interest.

## Author Contributions

S.H., F.L., and X.H. were involved in the conceptualization and design of the project. S.H., X.L., H.W., N.M., S.S., and A.B. performed experiments, and collected and analyzed the data. F.L. contributed to the generation of the mouse model and discussion of the project. S.H. wrote the first draft of the manuscript. S.H., S.Z., F.L., and X.H. contributed to data interpretation, data analysis and editing of the text. X.H. directed the project and provided laboratory resources for the study.

## Supporting information



Supporting Information

## Data Availability

All data that support the findings of this study are available from corresponding author upon reasonable request.

## References

[1] J. Y. Ho , Y. Franco , SSM Popul Health 2022, 17, 101052. 35242995 10.1016/j.ssmph.2022.101052 PMC8886050

[2] a) A. Griciuc , R. E. Tanzi , Curr. Opin. Neurol. 2021, 34, 228; 33560670 10.1097/WCO.0000000000000911 PMC7954128

[3] X. Chen , D. M. Holtzman , Immunity 2022, 55, 2236. 36351425 10.1016/j.immuni.2022.10.016 PMC9772134

[4] a) L. Sun , J. Wu , F. Du , X. Chen , Z. J. Chen , Science 2013, 339, 786; 23258413 10.1126/science.1232458 PMC3863629

[5] Q. Chen , L. Sun , Z. J. Chen , Nat. Immunol. 2016, 17, 1142. 27648547 10.1038/ni.3558

[6] H. Yang , H. Wang , J. Ren , Q. Chen , Z. J. Chen , Proc Natl Acad Sci U S A 2017, 114, E4612. 28533362 10.1073/pnas.1705499114 PMC5468617

[7] X. Gui , H. Yang , T. Li , X. Tan , P. Shi , M. Li , F. Du , Z. J. Chen , Nature 2019, 567, 262. 30842662 10.1038/s41586-019-1006-9 PMC9417302

[8] T. Li , Z. J. Chen , J. Exp. Med. 2018, 215, 1287. 29622565 10.1084/jem.20180139 PMC5940270

[9] H. Wang , S. Hu , X. Chen , H. Shi , C. Chen , L. Sun , Z. J. Chen , Proc Natl Acad Sci U S A 114, 1637. 10.1073/pnas.1621363114 PMC5320994 28137885

[10] L. Masanneck , S. Eichler , A. Vogelsang , M. Korsen , H. Wiendl , T. Budde , S. G. Meuth , Int. J. Mol. Sci. 2020, 21, 9249. 33291536 10.3390/ijms21239249 PMC7730283

[11] D. G. Standaert , G. M. Childers , Proc Natl Acad Sci U S A 2022, 119, e2204058119. 35446614 10.1073/pnas.2204058119 PMC9170025

[12] C. H. Yu , S. Davidson , C. R. Harapas , J. B. Hilton , M. J. Mlodzianoski , P. Laohamonthonkul , C. Louis , R. R. J. Low , J. Moecking , D. De Nardo , K. R. Balka , D. J. Calleja , F. Moghaddas , E. Ni , C. A. McLean , A. L. Samson , S. Tyebji , C. J. Tonkin , C. R. Bye , B. J. Turner , G. Pepin , M. P. Gantier , K. L. Rogers , K. McArthur , P. J. Crouch , S. L. Masters , Cell 2020, 183, 636. 33031745 10.1016/j.cell.2020.09.020 PMC7599077

[13] J. P. Barrett , S. M. Knoblach , S. Bhattacharya , H. Gordish‐Dressman , B. A. Stoica , D. J. Loane , Front Immunol 2021, 12, 710608. 34504493 10.3389/fimmu.2021.710608 PMC8423402

[14] Q. Li , Y. Cao , C. Dang , B. Han , R. Han , H. Ma , J. Hao , L. Wang , EMBO Mol. Med. 2020, 12, 11002. 10.15252/emmm.201911002 PMC7136961 32239625

[15] N. S. Muhammet , F. Gulen , A. Keller , M. Schwabenland , C. Liu , S. Glück , V V. Thacker , L. Favre , B. Mangeat , L J. Kroese , P. Krimpenfort , M. Prinz , A. Ablasser , Nature 2023, 620, 374. 37532932 10.1038/s41586-023-06373-1 PMC10412454

[16] a) X. Xie , G. Ma , X. Li , J. Zhao , Z. Zhao , J. Zeng , Nat Aging 2023, 3, 202; 37118112 10.1038/s43587-022-00337-2

[17] a) Y. Chen , D. M. Holtzman , Trends Immunol. 2024, 45, 768; 39278789 10.1016/j.it.2024.08.001

[18] A. F. Abdel‐Magid , ACS Med. Chem. Lett. 2024, 15, 1424. 39291025 10.1021/acsmedchemlett.4c00392 PMC11403758

[19] A. Mullard , Nat Rev Drug Discov 2023, 22, 939. 37949966 10.1038/d41573-023-00185-8

[20] a) L. Lama , C. Adura , W. Xie , D. Tomita , T. Kamei , V. Kuryavyi , T. Gogakos , J. I. Steinberg , M. Miller , L. Ramos‐Espiritu , Y. Asano , S. Hashizume , J. Aida , T. Imaeda , R. Okamoto , A. J. Jennings , M. Michino , T. Kuroita , A. Stamford , P. Gao , P. Meinke , J. F. Glickman , D. J. Patel , T. Tuschl , Nat. Commun. 2019, 10, 2261; 31113940 10.1038/s41467-019-08620-4 PMC6529454

[21] X. Wang , C. Yang , X. Wang , J. Miao , W. Chen , Y. Zhou , Y. Xu , Y. An , A. Cheng , W. Ye , M. Chen , D. Song , X. Yuan , J. Wang , P. Qian , A. R. Wu , Z. Y. Zhang , K. Liu , Neuron 2023, 111, 236. 36370710 10.1016/j.neuron.2022.10.028 PMC9851977

[22] M. Sharma , S. Rajendrarao , N. Shahani , U. N. Ramirez‐Jarquin , S. Subramaniam , Proc Natl Acad Sci U S A 2020, 117, 15989. 32581130 10.1073/pnas.2002144117 PMC7354937

[23] A. M. Jeffries , I. Marriott , Neurosci. Lett. 2017, 658, 53. 28830822 10.1016/j.neulet.2017.08.039 PMC5645252

[24] H. Yu , K. Liao , Y. Hu , D. Lv , M. Luo , Q. Liu , L. Huang , S. Luo , Aging Dis 2022, 13, 1901. 36465181 10.14336/AD.2022.0316 PMC9662267

[25] a) Y. Zhang , K. Chen , S. A. Sloan , M. L. Bennett , A. R. Scholze , S. O'Keeffe , H. P. Phatnani , P. Guarnieri , C. Caneda , N. Ruderisch , S. Deng , S. A. Liddelow , C. Zhang , R. Daneman , T. Maniatis , B. A. Barres , J. Q. Wu , J. Neurosci. 2014, 34, 11929; 25186741 10.1523/JNEUROSCI.1860-14.2014 PMC4152602

[26] D. E. Russ , R. B. P. Cross , L. Li , S. C. Koch , K. J. E. Matson , A. Yadav , M. R. Alkaslasi , D. I. Lee , C. E. Le Pichon , V. Menon , A. J. Levine , Nat. Commun. 2021, 12, 5722. 34588430 10.1038/s41467-021-25125-1 PMC8481483

[27] E. Spangenberg , P. L. Severson , L. A. Hohsfield , J. Crapser , J. Zhang , E. A. Burton , Y. Zhang , W. Spevak , J. Lin , N. Y. Phan , G. Habets , A. Rymar , G. Tsang , J. Walters , M. Nespi , P. Singh , S. Broome , P. Ibrahim , C. Zhang , G. Bollag , B. L. West , K. N. Green , Nat. Commun. 2019, 10, 3758. 31434879 10.1038/s41467-019-11674-z PMC6704256

[28] S. Thakur , R. Dhapola , P. Sarma , B. Medhi , D. H. Reddy , Inflammation 2023, 46, 1. 35986874 10.1007/s10753-022-01721-1

[29] A. Deczkowska , H. Keren‐Shaul , A. Weiner , M. Colonna , M. Schwartz , I. Amit , Cell 2018, 173, 1073. 29775591 10.1016/j.cell.2018.05.003

[30] P. d'Errico , S. Ziegler‐Waldkirch , V. Aires , P. Hoffmann , C. Mezo , D. Erny , L. S. Monasor , S. Liebscher , V. M. Ravi , K. Joseph , O. Schnell , K. Kierdorf , O. Staszewski , S. Tahirovic , M. Prinz , M. Meyer‐Luehmann , Nat. Neurosci. 2022, 25, 20. 34811521 10.1038/s41593-021-00951-0 PMC8737330

[31] C. Venegas , S. Kumar , B. S. Franklin , T. Dierkes , R. Brinkschulte , D. Tejera , A. Vieira‐Saecker , S. Schwartz , F. Santarelli , M. P. Kummer , A. Griep , E. Gelpi , M. Beilharz , D. Riedel , D. T. Golenbock , M. Geyer , J. Walter , E. Latz , M. T. Heneka , Nature 2017, 552, 355. 29293211 10.1038/nature25158

[32] a) T. Masuda , M. Tsuda , R. Yoshinaga , H. Tozaki‐Saitoh , K. Ozato , T. Tamura , K. Inoue , Cell Rep. 2012, 1, 334; 22832225 10.1016/j.celrep.2012.02.014 PMC4158926

[33] R. B. Knowles , C. Wyart , S. V. Buldyrev , L. Cruz , B. Urbanc , M. E. Hasselmo , H. E. Stanley , B. T. Hyman , Proc Natl Acad Sci U S A 1999, 96, 5274. 10220456 10.1073/pnas.96.9.5274 PMC21854

[34] K. R. Sadleir , P. C. Kandalepas , V. Buggia‐Prevot , D. A. Nicholson , G. Thinakaran , R. Vassar , Acta Neuropathol. 2016, 132, 235. 26993139 10.1007/s00401-016-1558-9 PMC4947125

[35] C. Condello , P. Yuan , A. Schain , J. Grutzendler , Nat. Commun. 2015, 6, 6176. 25630253 10.1038/ncomms7176 PMC4311408

[36] Y. Zhou , W. M. Song , P. S. Andhey , A. Swain , T. Levy , K. R. Miller , P. L. Poliani , M. Cominelli , S. Grover , S. Gilfillan , M. Cella , T. K. Ulland , K. Zaitsev , A. Miyashita , T. Ikeuchi , M. Sainouchi , A. Kakita , D. A. Bennett , J. A. Schneider , M. R. Nichols , S. A. Beausoleil , J. D. Ulrich , D. M. Holtzman , M. N. Artyomov , M. Colonna , Nat. Med. 2020, 26, 131. 31932797 10.1038/s41591-019-0695-9 PMC6980793

[37] S. Jin , C. F. Guerrero‐Juarez , L. Zhang , I. Chang , R. Ramos , C. H. Kuan , P. Myung , M. V. Plikus , Q. Nie , Nat. Commun. 2021, 12, 1088. 33597522 10.1038/s41467-021-21246-9 PMC7889871

[38] R. Mi , W. Chen , A. Hoke , Proc Natl Acad Sci U S A 2007, 104, 4664. 17360581 10.1073/pnas.0603243104 PMC1838658

[39] Z. Liu , M. Yan , W. Lei , R. Jiang , W. Dai , J. Chen , C. Wang , L. Li , M. Wu , X. Nian , D. Li , D. Sun , X. Lv , C. Wang , C. Xie , L. Yao , C. Wu , J. Hu , N. Xiao , W. Mo , Z. Wang , L. Zhang , J. Clin. Invest. 2022, 132, e155096. 35143418 10.1172/JCI155096 PMC8970680

[40] a) T. Allison , J. Langerman , S. Sabri , M. Otero‐Garcia , A. Lund , J. Huang , X. Wei , R. A. Samarasinghe , D. Polioudakis , I. Mody , I. Cobos , B. G. Novitch , D. H. Geschwind , K. Plath , W. E. Lowry , Stem Cell Rep. 2021, 16, 2548; 10.1016/j.stemcr.2021.08.006 PMC8514853 34506726

[41] T. H. Ferreira‐Vieira , I. M. Guimaraes , F. R. Silva , F. M. Ribeiro , Curr. Neuropharmacol. 2016, 14, 101. 26813123 10.2174/1570159X13666150716165726 PMC4787279

[42] A. Serrano‐Pozo , M. P. Frosch , E. Masliah , B. T. Hyman , Cold Spring Harb Perspect Med 2011, 1, a006189. 22229116 10.1101/cshperspect.a006189 PMC3234452

[43] a) J. Hardy , D. Allsop , Trends Pharmacol. Sci. 1991, 12, 383; 1763432 10.1016/0165-6147(91)90609-v

[44] C. Y. Lee , G. E. Landreth , J Neural Transm (Vienna) 2010, 117, 949. 20552234 10.1007/s00702-010-0433-4 PMC3653296

[45] a) C. Haass , A. Y. Hung , D. J. Selkoe , J. Neurosci. 1991, 11, 3783; 1744690 10.1523/JNEUROSCI.11-12-03783.1991 PMC6575298

[46] S. K Shabestari , S. Morabito , E. P. Danhash , A. McQuade , J. R. Sanchez , E. Miyoshi , J. P. Chadarevian , C. Claes , M. A. Coburn , J. Hasselmann , J. Hidalgo , K. N. Tran , A. C. Martini , W. Chang Rothermich , J. Pascual , E. Head , D. A. Hume , C. Pridans , H. Davtyan , V. Swarup , M. Blurton‐Jones , Cell Rep. 2022, 39, 110961. 35705056 10.1016/j.celrep.2022.110961 PMC9285116

[47] a) T. R. Jay , C. M. Miller , P. J. Cheng , L. C. Graham , S. Bemiller , M. L. Broihier , G. Xu , D. Margevicius , J. C. Karlo , G. L. Sousa , A. C. Cotleur , O. Butovsky , L. Bekris , S. M. Staugaitis , J. B. Leverenz , S. W. Pimplikar , G. E. Landreth , G. R. Howell , R. M. Ransohoff , B. T. Lamb , J. Exp. Med. 2015, 212, 287; 25732305 10.1084/jem.20142322 PMC4354365

[48] a) N. F. Fitz , C. M. Wolfe , B. E. Playso , R. J. Biedrzycki , Y. Lu , K. N. Nam , I. Lefterov , R. Koldamova , Mol Neurodegener 2020, 15, 41; 32703241 10.1186/s13024-020-00394-4 PMC7379780

[49] T. R. Jay , A. M. Hirsch , M. L. Broihier , C. M. Miller , L. E. Neilson , R. M. Ransohoff , B. T. Lamb , G. E. Landreth , J. Neurosci. 2017, 37, 637. 28100745 10.1523/JNEUROSCI.2110-16.2016 PMC5242410

[50] S. M. Bemiller , T. J. McCray , K. Allan , S. V. Formica , G. Xu , G. Wilson , O. N. Kokiko‐Cochran , S. D. Crish , C. A. Lasagna‐Reeves , R. M. Ransohoff , G. E. Landreth , B. T. Lamb , Mol Neurodegener 2017, 12, 74. 29037207 10.1186/s13024-017-0216-6 PMC5644120

[51] N. Zhao , W. Qiao , F. Li , Y. Ren , J. Zheng , Y. A. Martens , X. Wang , L. Li , C. C. Liu , K. Chen , Y. Zhu , T. C. Ikezu , Z. Li , A. D. Meneses , Y. Jin , J. A. Knight , Y. Chen , L. Bastea , C. Linares , B. Sonustun , L. Job , M. L. Smith , M. Xie , Y. U. Liu , A. D. Umpierre , K. Haruwaka , Z. S. Quicksall , P. Storz , Y. W. Asmann , L. J. Wu , et al., J. Exp. Med. 2022, 219, e20212479. 36107206 10.1084/jem.20212479 PMC9481739

[52] L. Unterholzner , G. Dunphy , Mol Cell Oncol 2019, 6, 1558682. 31211228 10.1080/23723556.2018.1558682 PMC6548478

[53] S. Chung , J. H. Jeong , J. C. Park , J. W. Han , Y. Lee , J. I. Kim , I. Mook‐Jung , Exp. Mol. Med. 2024, 56, 1936. 39218977 10.1038/s12276-024-01295-y PMC11447230

[54] a) M. Sochocka , B. S. Diniz , J. Leszek , Mol. Neurobiol. 2017, 54, 8071; 27889895 10.1007/s12035-016-0297-1 PMC5684251

[55] S. E. Hickman , E. K. Allison , J. El Khoury , J. Neurosci. 2008, 28, 8354. 18701698 10.1523/JNEUROSCI.0616-08.2008 PMC2597474

[56] S. H. Baik , S. Kang , S. M. Son , I. Mook‐Jung , Glia 2016, 64, 2274. 27658617 10.1002/glia.23074

[57] N. Mittra , S. He , H. Bao , A. Bhattacharjee , S. G. Dodds , J. L. Dupree , X. Han , bioRxiv 2024, 10.1101/2024.11.14.623651. PMC12789058 41513633

[58] a) X. Han , D. M. Holtzman , D. W. McKeel, Jr. , J. Kelley , J. C. Morris , J. Neurochem. 2002, 82, 809; 12358786 10.1046/j.1471-4159.2002.00997.x

[59] F. Leng , P. Edison , Nat. Rev. Neurol. 2021, 17, 157. 33318676 10.1038/s41582-020-00435-y

[60] a) H. Li , L. Xu , W. Jiang , X. Qiu , H. Xu , F. Zhu , Y. Hu , S. Liang , C. Cai , W. Qiu , Z. Lu , Y. Cui , C. Tang , Cell Rep. 2023, 42, 113022; 37610873 10.1016/j.celrep.2023.113022

[61] C. H. Andrade‐Moraes , A. V. Oliveira‐Pinto , E. Castro‐Fonseca , C. G. da Silva , D. M. Guimaraes , D. Szczupak , D. R. Parente‐Bruno , L. R. Carvalho , L. Polichiso , B. V. Gomes , L. M. Oliveira , R. D. Rodriguez , R. E. Leite , R. E. Ferretti‐Rebustini , W. Jacob‐Filho , C. A. Pasqualucci , L. T. Grinberg , R. Lent , Brain 2013, 136, 3738. 24136825 10.1093/brain/awt273 PMC3859218

[62] S. Burden , Y. Yarden , Neuron 1997, 18, 847. 9208852 10.1016/s0896-6273(00)80324-4

[63] S. A. Wolf , H. W. Boddeke , H. Kettenmann , Annu. Rev. Physiol. 2017, 79, 619. 27959620 10.1146/annurev-physiol-022516-034406

[64] D. P. Schafer , E. K. Lehrman , B. Stevens , Glia 2013, 61, 24. 22829357 10.1002/glia.22389 PMC4082974

[65] S. Hong , V. F. Beja‐Glasser , B. M. Nfonoyim , A. Frouin , S. Li , S. Ramakrishnan , K. M. Merry , Q. Shi , A. Rosenthal , B. A. Barres , C. A. Lemere , D. J. Selkoe , B. Stevens , Science 2016, 352, 712. 27033548 10.1126/science.aad8373 PMC5094372

[66] D. K. Wilton , L. Dissing‐Olesen , B. Stevens , Annu. Rev. Neurosci. 2019, 42, 107. 31283900 10.1146/annurev-neuro-070918-050306

[67] a) S. Forner , S. Kawauchi , G. Balderrama‐Gutierrez , E. A. Kramar , D. P. Matheos , J. Phan , D. I. Javonillo , K. M. Tran , E. Hingco , C. da Cunha , N. Rezaie , J. A. Alcantara , D. Baglietto‐Vargas , C. Jansen , J. Neumann , M. A. Wood , G. R. MacGregor , A. Mortazavi , A. J. Tenner , F. M. LaFerla , K. N. Green , Sci Data 2021, 8, 270; 34654824 10.1038/s41597-021-01054-y PMC8519958

[68] R. M. Deacon , Nat. Protoc. 2006, 1, 1117. 17406392 10.1038/nprot.2006.170

[69] K. L. Youmans , S. Leung , J. Zhang , E. Maus , K. Baysac , G. Bu , R. Vassar , C. Yu , M. J. LaDu , J. Neurosci. Methods 2011, 196, 51. 21219931 10.1016/j.jneumeth.2010.12.025 PMC3049315

[70] I. C. Clark , K. M. Fontanez , R. H. Meltzer , Y. Xue , C. Hayford , A. May‐Zhang , C. D'Amato , A. Osman , J. Q. Zhang , P. Hettige , J. S. A. Ishibashi , C. L. Delley , D. W. Weisgerber , J. M. Replogle , M. Jost , K. T. Phong , V. E. Kennedy , C. A. C. Peretz , E. A. Kim , S. Song , W. Karlon , J. S. Weissman , C. C. Smith , Z. J. Gartner , A. R. Abate , Nat. Biotechnol. 2023, 41, 1557. 36879006 10.1038/s41587-023-01685-z PMC10635830

[71] Y. Hao , S. Hao , E. Andersen‐Nissen , W. M. Mauck, 3rd , S. Zheng , A. Butler , M. J. Lee , A. J. Wilk , C. Darby , M. Zager , P. Hoffman , M. Stoeckius , E. Papalexi , E. P. Mimitou , J. Jain , A. Srivastava , T. Stuart , L. M. Fleming , B. Yeung , A. J. Rogers , J. M. McElrath , C. A. Blish , R. Gottardo , P. Smibert , R. Satija , Cell 2021, 184, 3573. 34062119 10.1016/j.cell.2021.04.048 PMC8238499

[72] C. S. McGinnis , L. M. Murrow , Z. J. Gartner , Cell Syst 2019, 8, 329. 30954475 10.1016/j.cels.2019.03.003 PMC6853612

[73] G. Finak , A. McDavid , M. Yajima , J. Deng , V. Gersuk , A. K. Shalek , C. K. Slichter , H. W. Miller , M. J. McElrath , M. Prlic , P. S. Linsley , R. Gottardo , Genome Biol. 2015, 16, 278. 26653891 10.1186/s13059-015-0844-5 PMC4676162

