# Supplementary material for: Microglial cGAS Deletion Preserves Intercellular Communication and Alleviates Amyloid‐β‐Induced Pathogenesis of Alzheimer's Disease
Source: Adv Sci (Weinh). 2025 Feb 5;12(12):2410910. doi: 10.1002/advs.202410910 (PMC11948024; doi:10.1002/advs.202410910)
Supplement: Supplementary file 1 — Supporting Information [file ADVS-12-2410910-s001.docx]

**Supplementary information**

**Microglial cGAS Deletion Preserves Intercellular Communication and Alleviates Amyloid-β-Induced Pathogenesis of Alzheimer's Disease**

Sijia He^1,2^, Xin Li^1^, Namrata Mittra^1^, Anindita Bhattacharjee^1^, Hu Wang^1^, Shujie Song^1^, Shangang Zhao^1,3^, Feng Liu^4,*^, and Xianlin Han^1,5,*^

^1^Barshop Institute for Longevity and Aging Studies, University of Texas Health Science Center at San Antonio, San Antonio, TX 78229, USA. ^2^Department of Cellular and Integrative Physiology, University of Texas Health Science Center at San Antonio, San Antonio, TX 78229, USA. ^3^Division of Endocrinology, Department of Medicine, University of Texas Health Science Center at San Antonio, San Antonio, TX 78229, USA. ^4^Metabolic Syndrome Research Center, The Second Xiangya Hospital of Central South University, Changsha, Hunan 410011, China. ^5^Division of Diabetes, Department of Medicine, University of Texas Health Science Center at San Antonio, San Antonio, TX 78229, USA.

***Corresponding authors:** Feng Liu, Email: [liuf001@csu.edu.cn;](mailto:liuf001@csu.edu.cn;)

Xianlin Han, Email: [hanx@uthscsa.edu](mailto:hanx@uthscsa.edu);

**This file includes:**

Supporting text

Figures S1 to S9

Tables S1 to S2


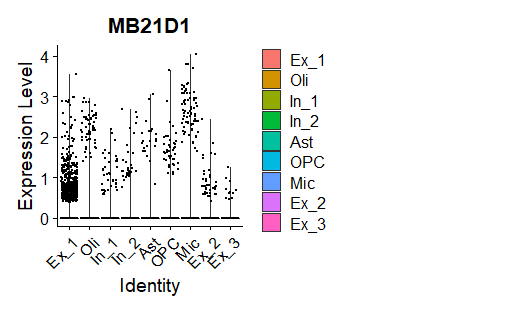


**a.**

**b.**

**c.**


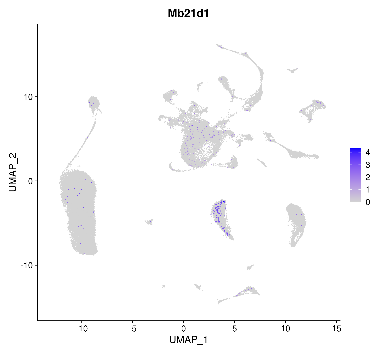


**MB21D1**

Microglia

Oligodendrocyte

Neuron

Astrocytes

Endothelial cells


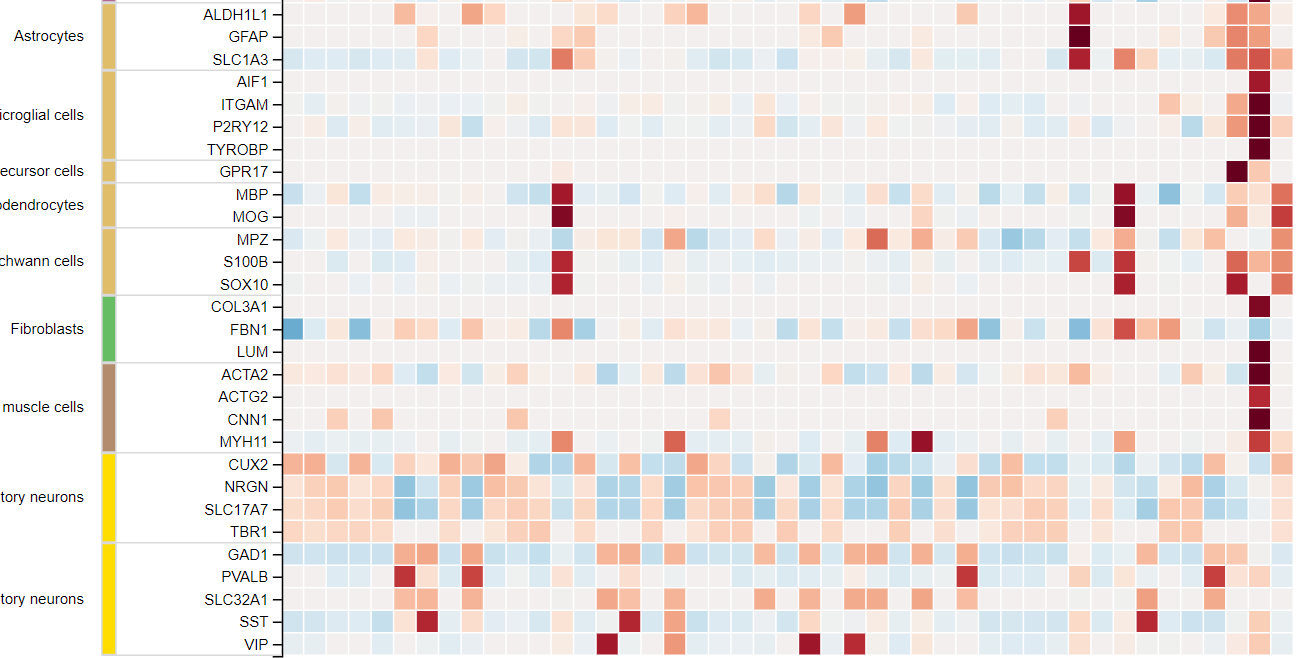

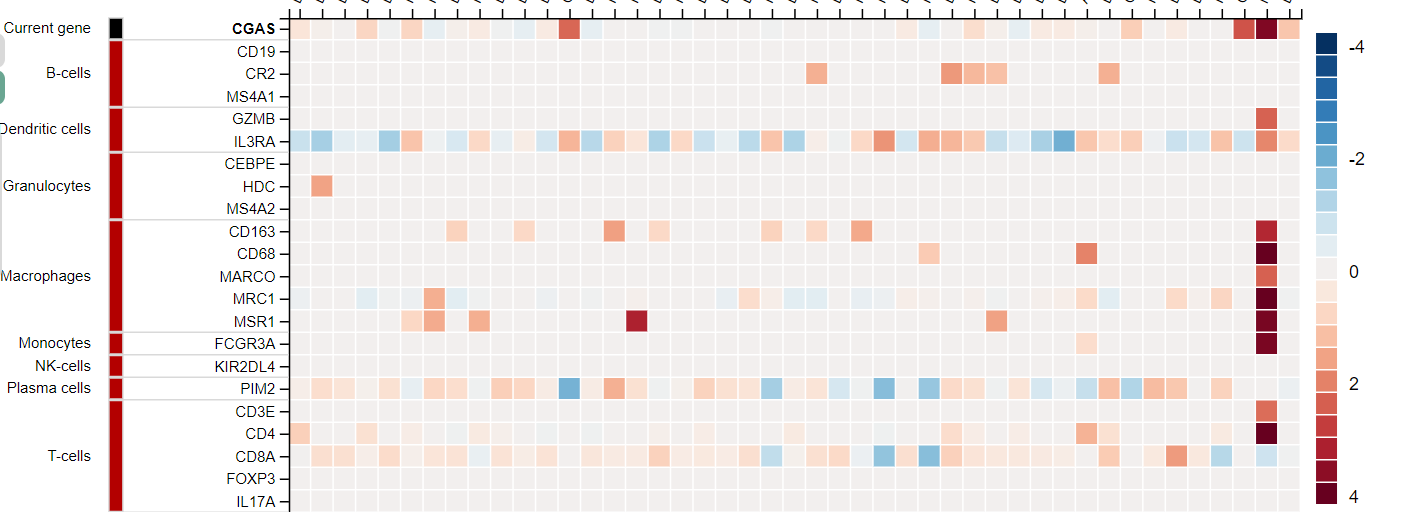

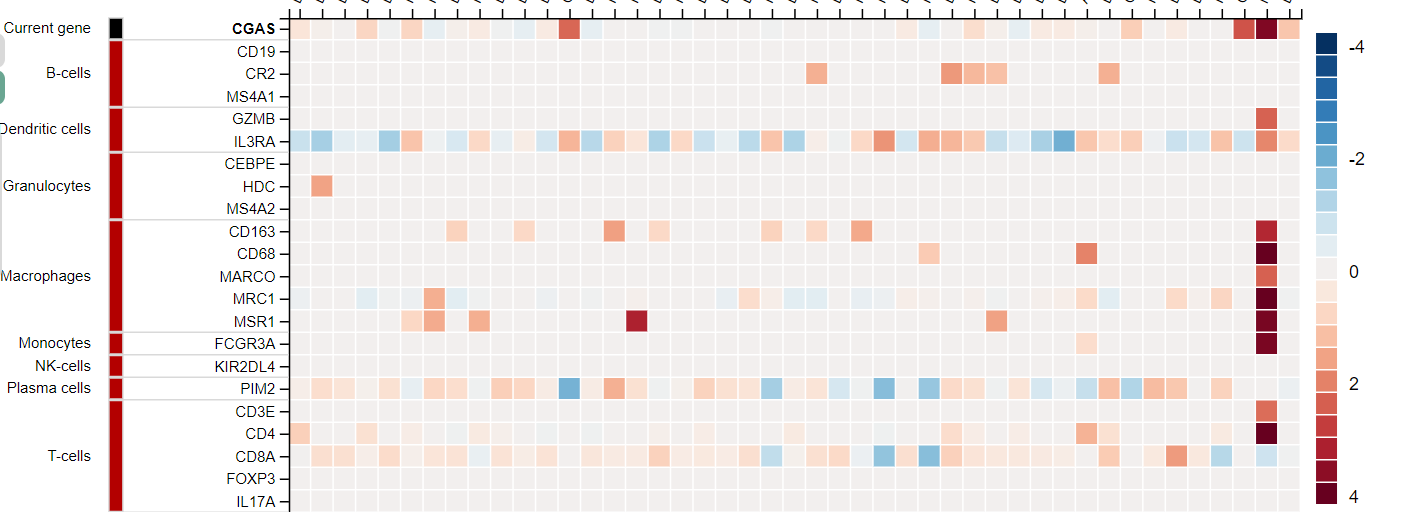

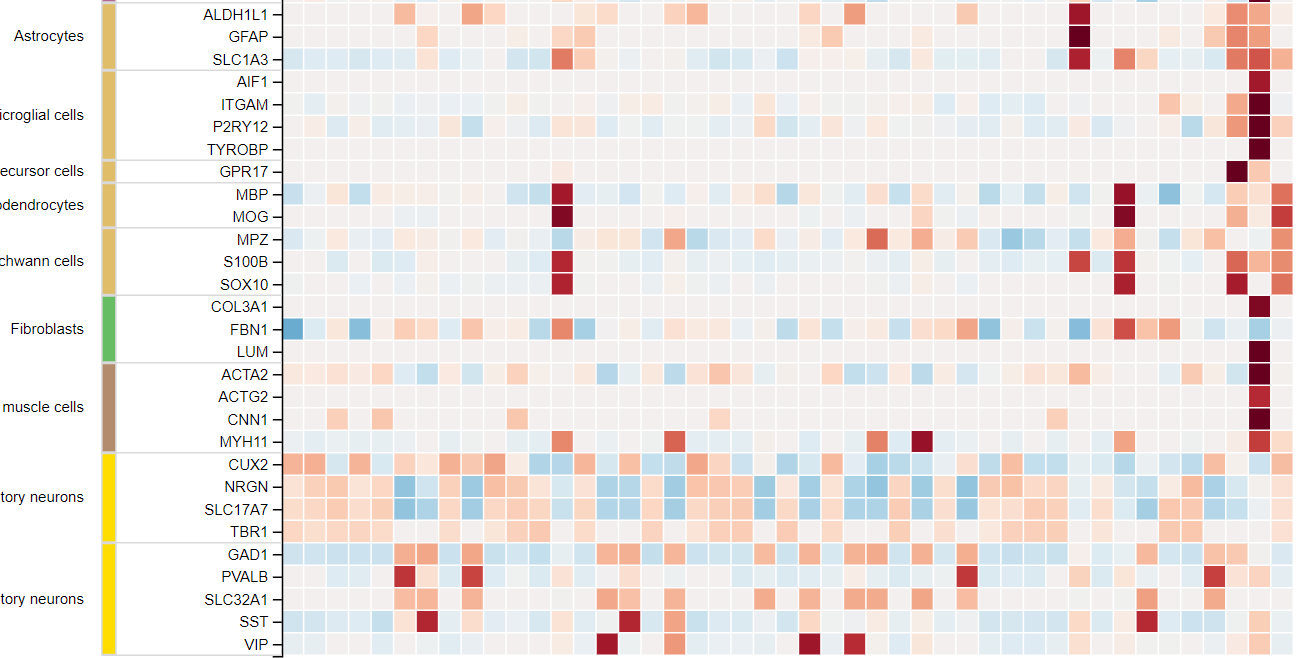


Oligodendrocytes

Oligodendrocytes

OPC

Microglia

Neuron

Neuron

Astrocyte

Oli.

OPC

Neuron

Microglia

Astrocytes

**cGAS**

**d.**

**Figure S1. cGAS expression in human brain and mouse spinal cord tissues.** a) RNA-Seq evaluation of cGAS expression levels in different cell types of human brain (samples were cells isolated from temporal lobe cortex region), data downloaded from (<https://www.brainrnaseq.org/>). Reproduced with permission (DIO: 10.1016/j.neuron.2015.11.013). b) cGAS expression in human brain measured by single cell sequencing (samples were from primary motor cortex region). Data adapted from “Human Protein Atlas proteinatlas.org”. Reproduced with permission (DIO: 10.1126/science.1260419). c) cGAS mRNA levels in different cell populations measured by single-nucleus sequencing (samples were from prefrontal cortex region). Reproduced with permission (DIO: 10.1038/s41586-019-1195-2). Note that Ex-1 neuron was the most enriched cell population, while microglia had the highest cGAS levels. d) Single cell sequencing database inquiry of cGAS expression in mouse spinal cord tissues (samples were from lumbar spinal cord region). Data adapted from (https://seqseek.ninds.nih.gov/spinalcordinjury). Reproduced with permission (DIO: 10.1038/s41467-021-25125-1).


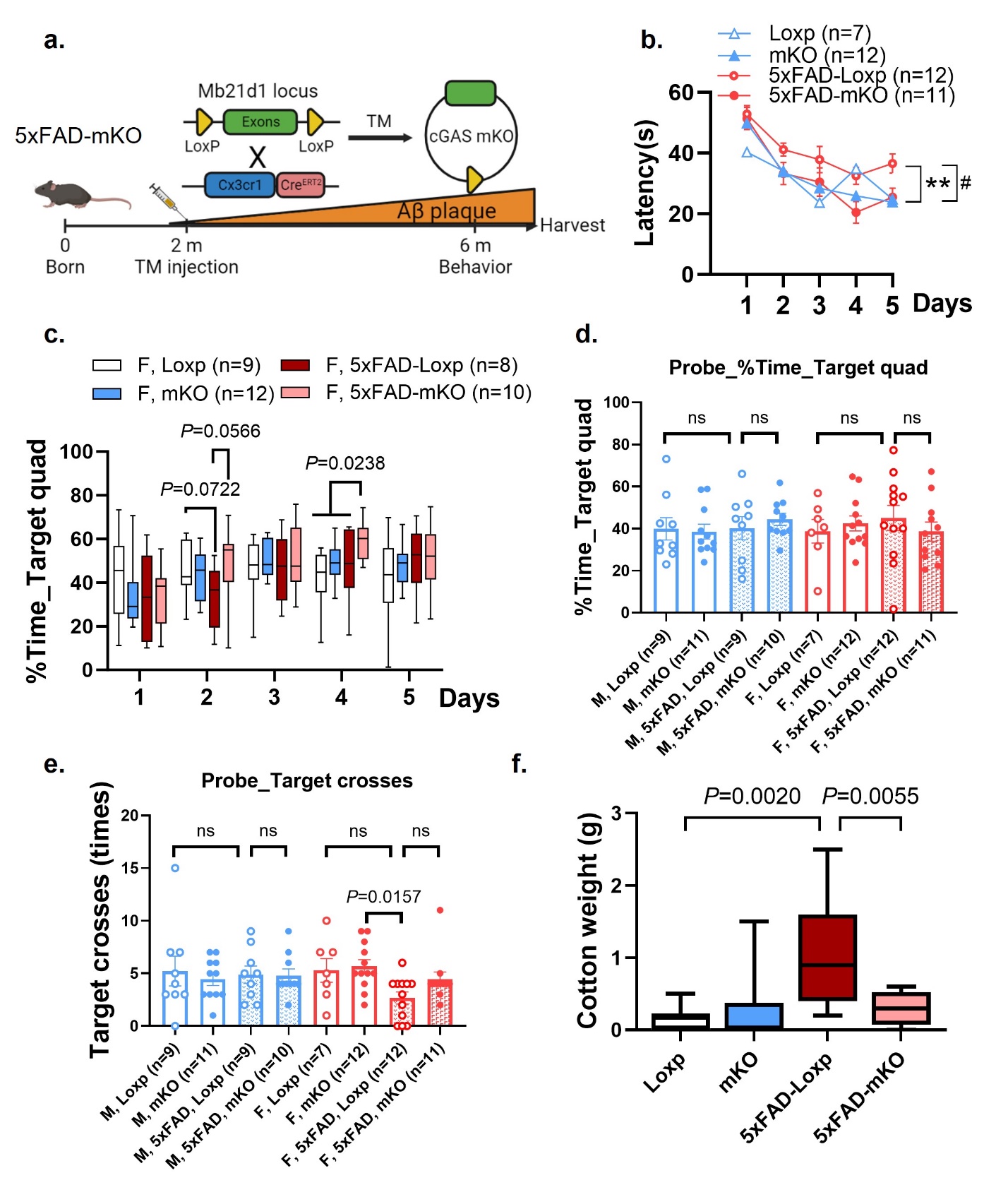


**Figure S2. Behavioral phenotypes of microglia-specific *cGAS* knockout mice.** a) Schematic illustration of the strategy for establishing the *cGAS* mKO mouse model. (Figure created using BioRender). b) Latency (^#^*P*=0.0149, ***P*=0.0038), and c) Percent time in the target quadrant during the training phase in an MWM test. (Showing female data from 6 months of age, *n*=8-12/genotype). d) Percent time in the target quadrant, and e) Times of target cross during probe test on the 6th day of MWM test. f) Weight of un-shredded cotton after a nest building test. (Male and female, 7 months old, *n*=8-12/group). Repeated measure analysis for b). Two-way ANOVA followed with Šidák correction for c), d), e) and f). *P* values are shown in each graph.


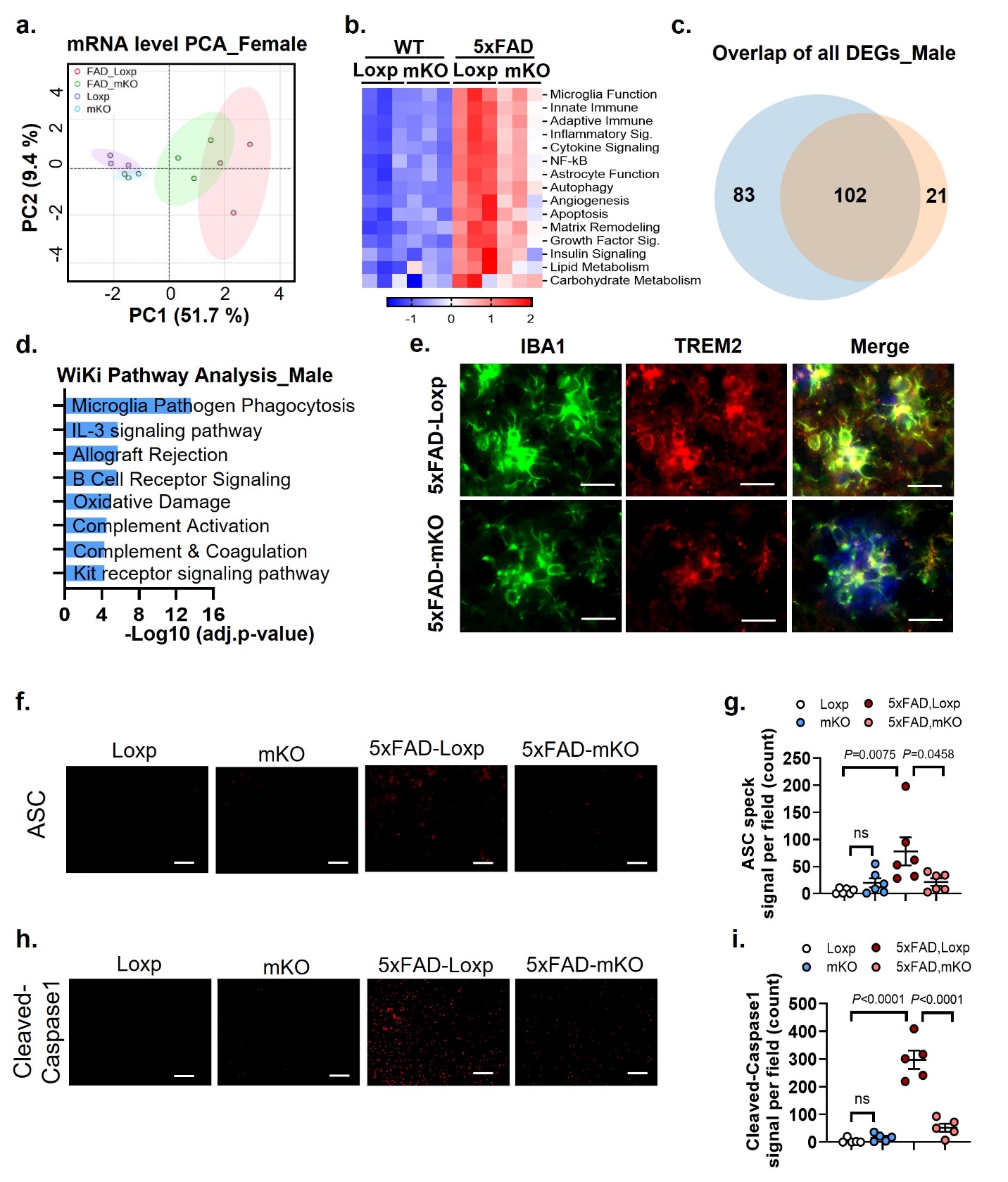


**Figure S3. Gene profile and inflammasome activity alterations in 5xFAD-mKO mice.** a) PCA analysis based on cerebrum tissue mRNA levels gathered from NanoString neuroinflammation panel. (*n*=3/group, female, 7 months old). b) Heatmap displaying pathway scores calculated using female NanoString data. c) Venn diagram showing overlap of DEGs that were altered by Aβ pathology and by cGAS deletion in male mice (note this includes both increased and decreased genes). d) WiKi Pathway analysis using 72 overlapped genes from Fig.4e. e) Representative IF staining of DAM marker TREM2 with co-staining of IBA1 and Aβ (blue color). Scale bars represent 20μm. f) IF evaluation of ASC levels in the cortex area of 7-month-old mice. g) Quantification of f), *n*=3 mice/group with 2 field pictures taken per mouse. h) IF evaluation of cleaved-Caspase1 levels in the cortex area of 7-month-old mice. i) Quantification of h), *n*=3 mice/group with 2 field pictures taken per mouse. Two-way ANOVA followed with Šidák correction for g) and i).


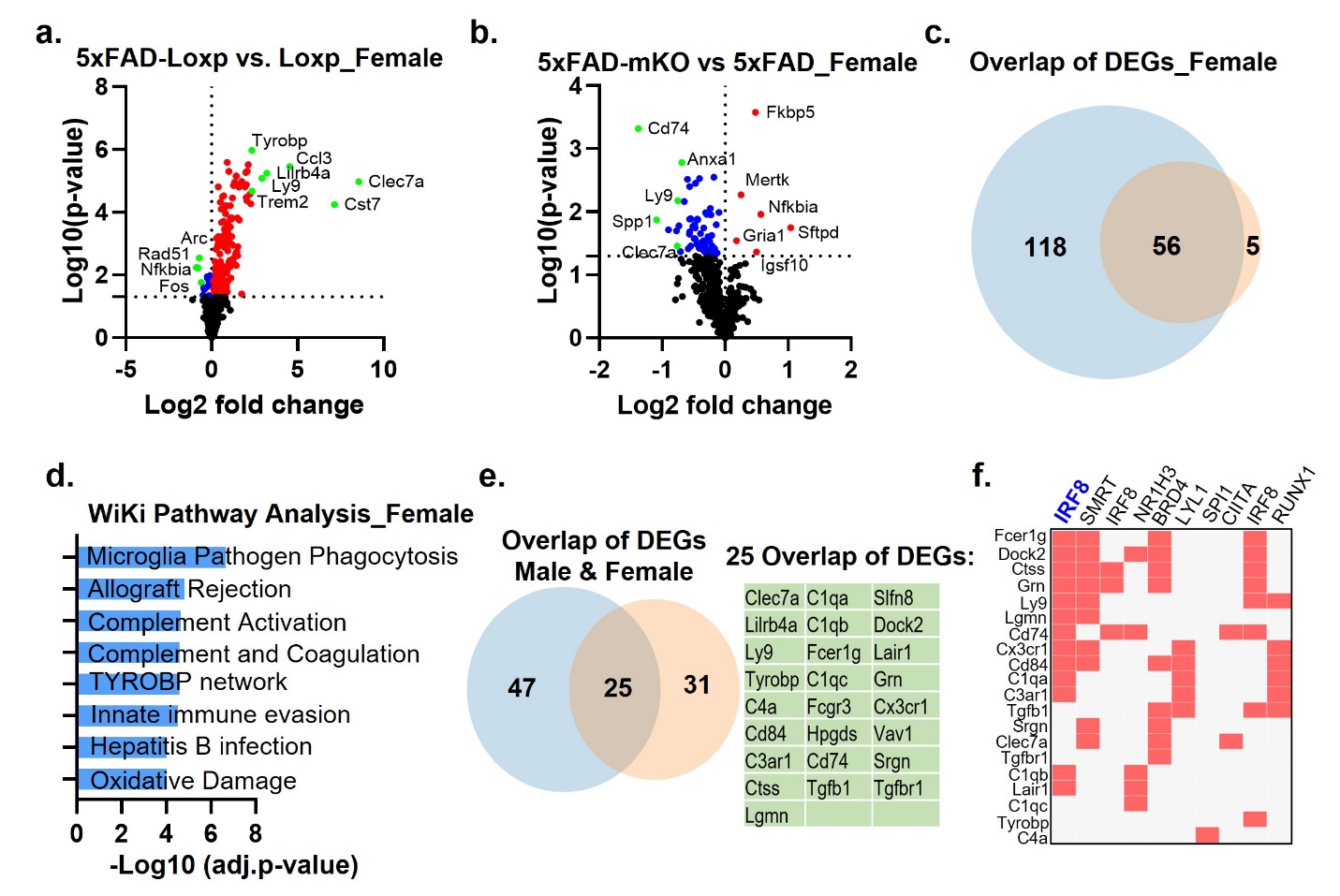


**Figure S4. Gene profile alterations in female 5xFAD-mKO mice.** a) Volcano plot showing female DEGs by comparing 5xFAD-Loxp to Loxp group. Red and blue color represents increased and decreased expression, respectively. Green color indicates genes of special interest. b) Volcano plot showing female DEGs by comparing 5xFAD-mKO to 5xFAD-Loxp group. c) Venn diagram showing overlapping female DEGs between plaque-induced genes (5xFAD-Loxp vs. Loxp, blue color) and genes decreased upon cGAS deletion (5xFAD-mKO vs. 5xFAD-Loxp, orange color) measured using NanoString (p-value ≤ 0.05). d) WiKi Pathway analysis using 56 overlapped genes from c). e) Venn diagram and list of the 25 overlapped DEGs that were induced by Aβ pathology and reduced by cGAS deletion in both sexes. f) Transcription factor analysis based on ChEA database using 25 overlapped gene from e).


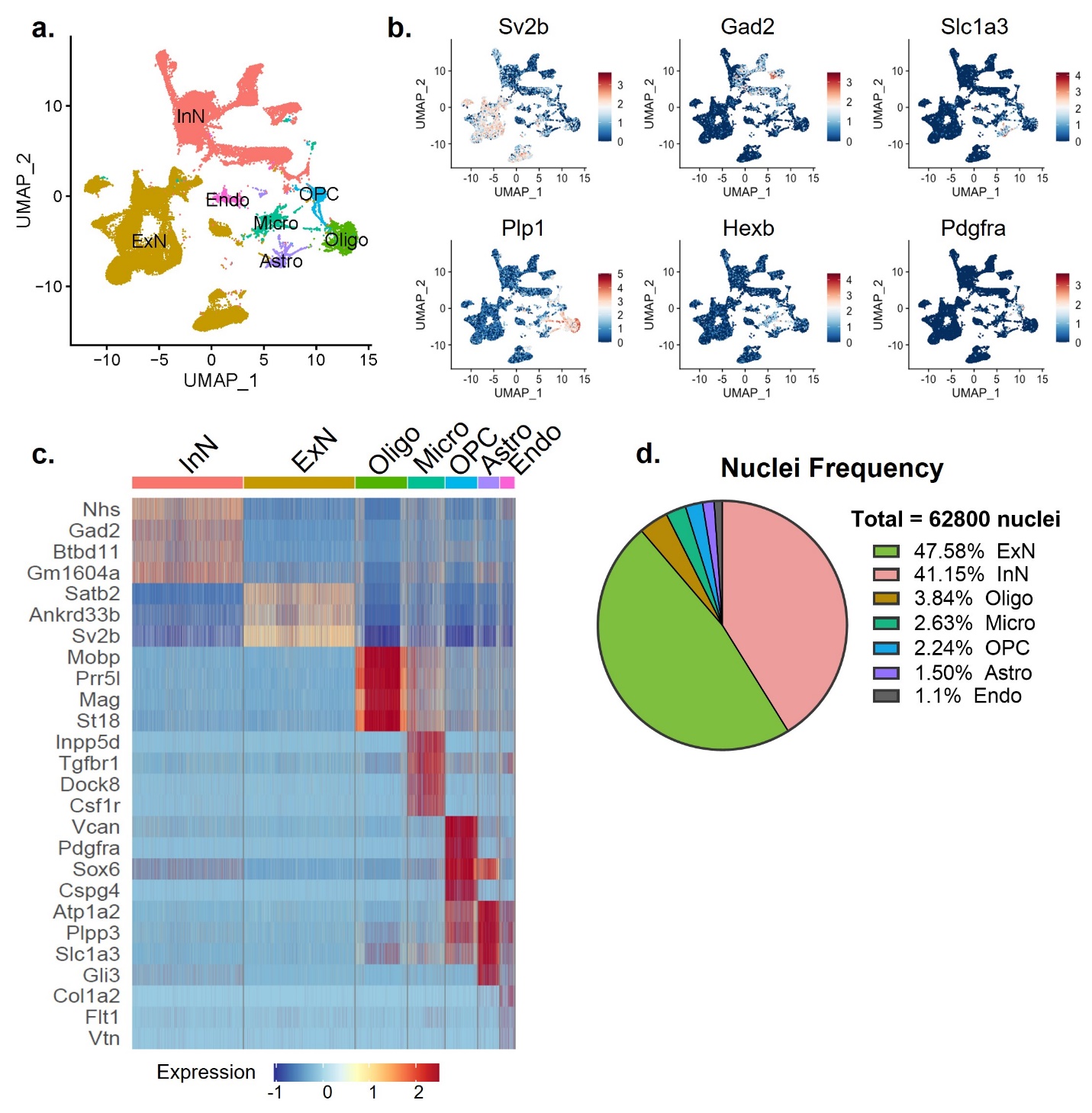


**Figure S5. Detection of major cell types in mouse cerebrum tissues by snRNA-seq.**  a) Uniform Manifold Approximation and Projection (UMAP) showing distinguished clusters representing 7 major cell populations in mouse brain (mix of all genotype groups). b) Feature plot evaluating the distribution of established cell type-specific markers. c) Heat map showing expression of specific markers identified from each cell population. d) Pie chart showing the frequency of each cell population across all genotypes. Mouse semi-cerebrum samples (n = 3) were pooled for sequencing of each genotype.

**
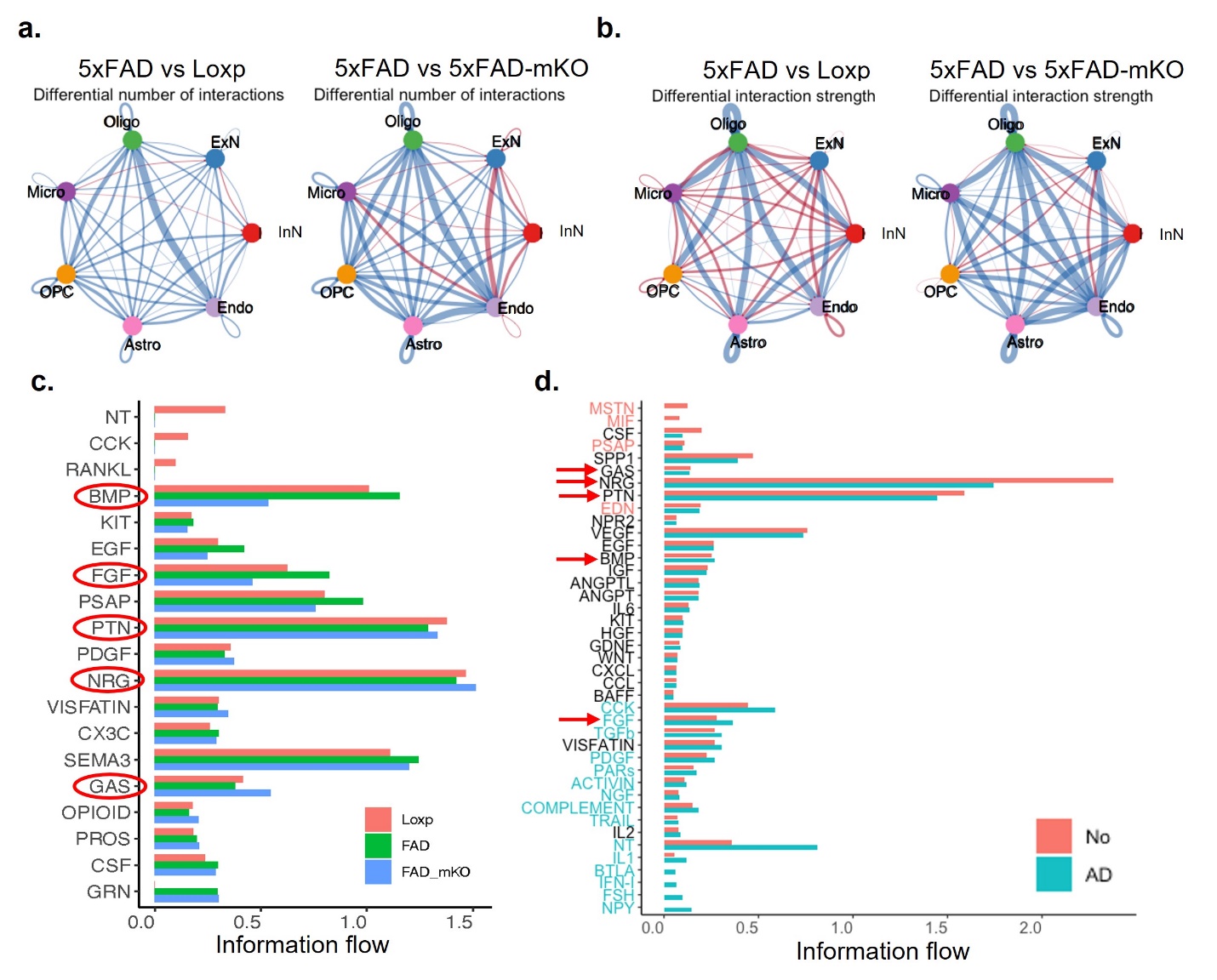
**

**Figure S6. CellChat evaluation of inter-cellular communication flow and strength.** a) Circle plot showing the differential number of interactions (blue color indicates decreased interaction, red color indicates increased interaction). b) Circle plot showing the differential strength of interactions (blue color indicates decreased interaction, red color indicates increased interaction). c) Overall interaction information flow contributed by each pathway in mice cerebrum. d) Overall interaction information flow contributed by each pathway in human brain (Analysis was performed based on data generated by ROSMAP single cell sequencing project. Reproduced with permission, DIO: 10.1038/s41586-019-1195-2).


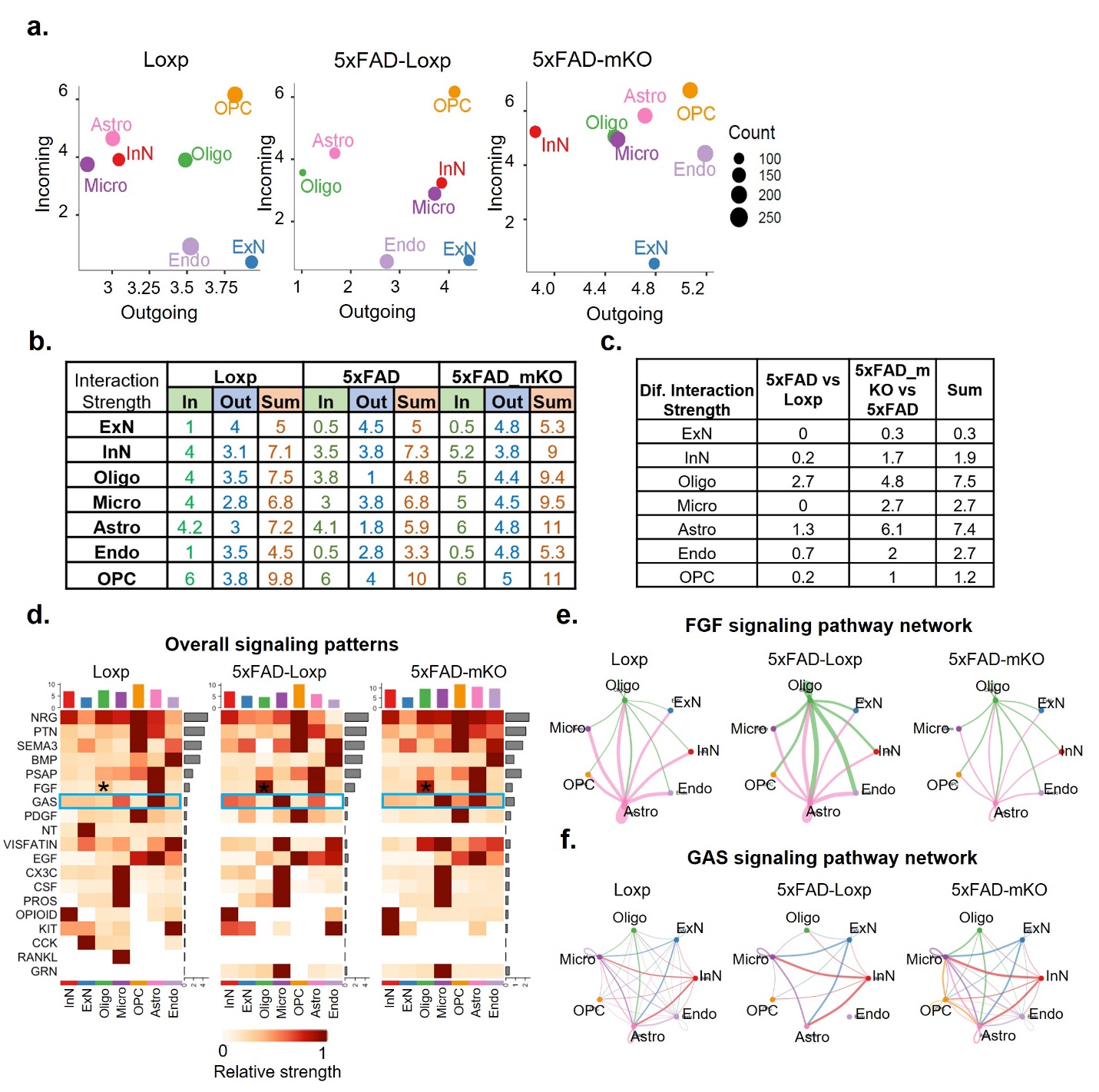


**Figure S7. CellChat evaluation of inter-cellular communication flow and strength based on cell types.** a) Scatter plot showing the incoming and outgoing interaction strength of each cell type from individual genotype groups. b) Interaction strength values associated with a). c) Group-wise comparison of interaction strength values in each cell type. d) Heatmap displaying the overall signaling patterns of each cell type from all 3 groups. e) Circle plot depicting the change of FGF and f) GAS signaling among different groups. Mouse semi-cerebrum samples (n = 3) were pooled for sequencing of each genotype.

**
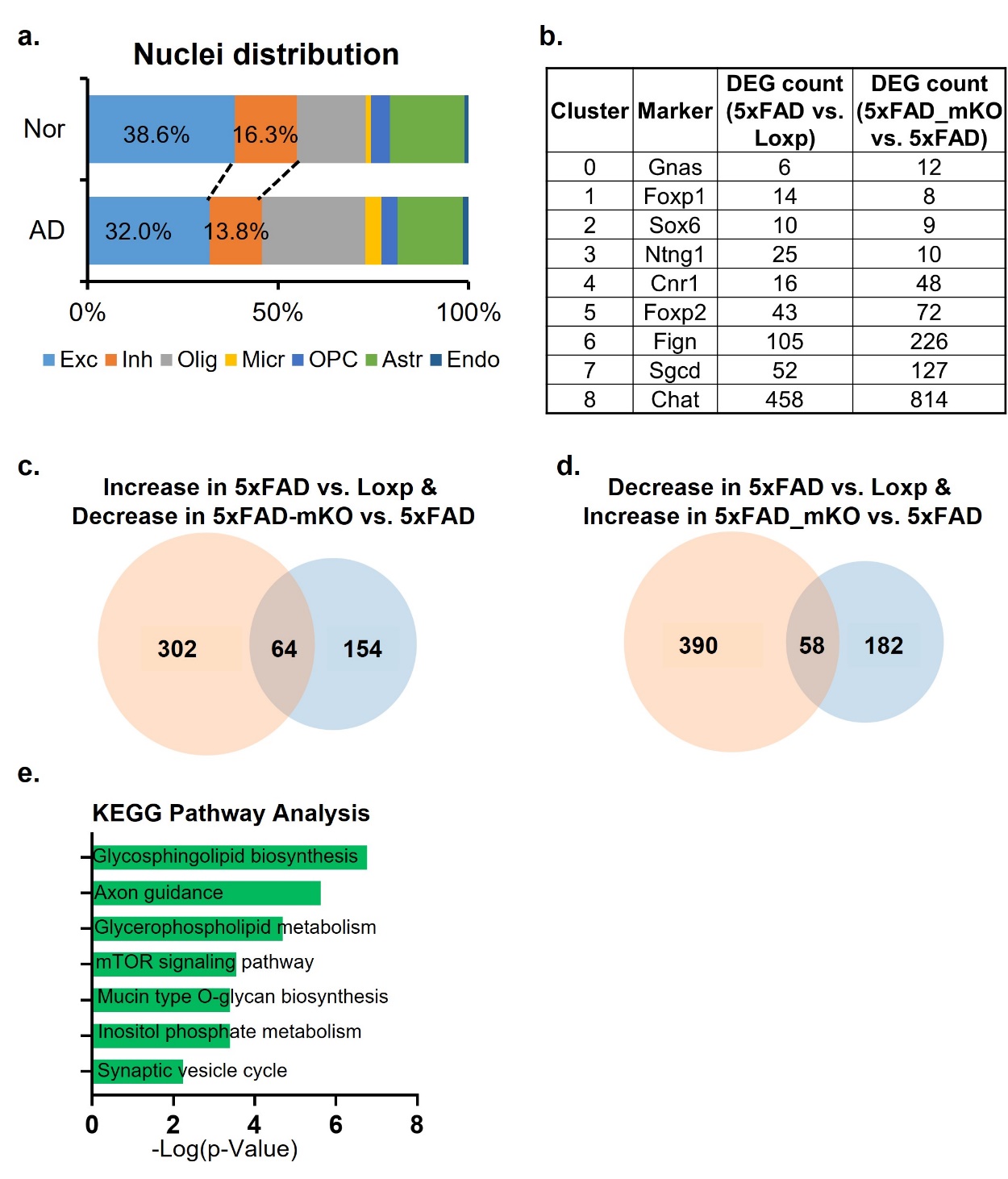
**

**Figure S8. Evaluation of neuronal cell population changes in AD and 5xFAD-mKO mice compared to the respective controls.** a) Alteration of cell frequency in AD brain vs Normal (Data generated by ROSMAP single cell sequencing project. Reproduced with permission, DIO: 10.1038/s41586-019-1195-2). b) The DEG count of each interneuron subclusters with respective comparisons as indicated in the chart. c) Venn diagram showing the overlap of genes that were increased in 5xFAD-Loxp vs. Loxp and decreased in 5xFAD-mKO vs. 5xFAD-Loxp in *Chat^+^* neuron population. d) Venn diagram showing the overlap of genes that were decreased in 5xFAD-Loxp vs. Loxp and increased in 5xFAD-mKO vs 5xFAD-Loxp in *Chat^+^* neuron population. e) KEGG pathway analysis using the list of shared genes that are oppositely regulated in the following two comparisons: 5xFAD-Loxp vs. Loxp, and 5xFAD-mKO vs 5xFAD-Loxp.


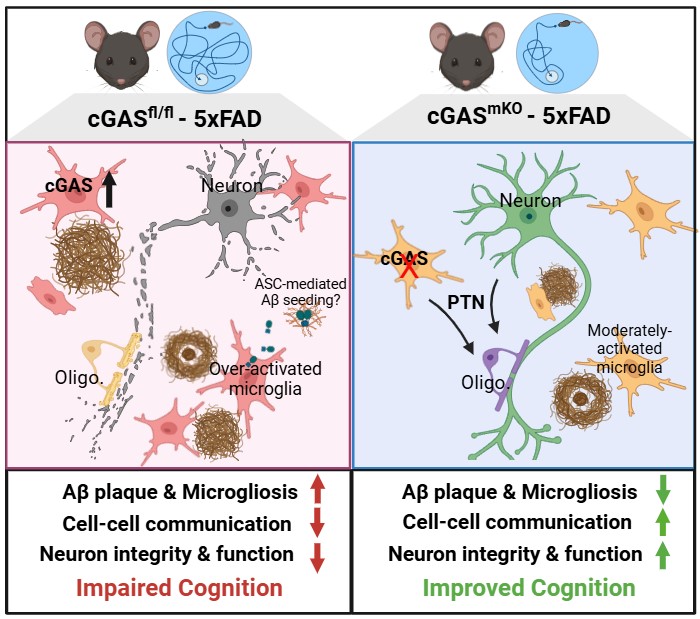


**Figure S9. Graphical summary of the impact of microglial cGAS deletion on AD brain.** Left panel illustrates that the elevated microglial cGAS expression in Aβ pathology promotes plaque-associated recruitment of over-activated microglia, loses the major cell-cell communication signaling (e.g., PTN), and accelerates neuronal loss, leading to cognitive dysfunction. Right panel illustrates that the deletion of microglial cGAS limits microglial reactivity, suppresses inflammasome activation, which potentially decreases ASC-mediated Aβ seeding effect, thus down-regulates plaque load. Further, the absence of microglial cGAS maintains cell-cell communication (e.g., PTN) and preserves myelin and neuronal integrity, leading to improved cognitive function. (Graph created using BioRender)

**Table S1. List of primers used**

| **Primer name** | **5'-sequence-3'** |
| --- | --- |
| **m-GAPDH_F** | GAGAAACCTGCCAAGTATG |
| **m-GAPDH_R** | GGAGTTGCTGTTGAAGTC |
| **m-cGas_F** | GGCAGCTACTATGAACATGTG |
| **m-cGas_R** | CTCAGCGGATTTCCTCGTGGAA |
| **m-IL1b-F** | GAAATGCCACCTTTTGACAGTG |
| **m-IL1b-R** | TGGATGCTCTCATCAGGACAG |
| **m-TNFα_F** | GCCTCTTCTCATTCCTGCTT |
| **m-TNFα_R** | CTCCTCCACTTGGTGGTTTG |
| **m-ChAT_F** | GAGCGAATCGTTGGTATGACAA |
| **m-ChAT_R** | AGGACGATGCCATCAAAAGG |
| **m-ChT1_F** | GCAGCTTTTGGGTGCCTG |
| **m-ChT1_R** | TGTGGAAGCTCCAATAGCTCC |
| **m-VAChT_F** | GGGTCGGCTCGGTCAATC |
| **m-VAChT_R** | CAAATAGCACGCCTATCTTCACAT |
| **h-GAPDH_F** | CATGTTCCAATATGATTCCACC |
| **h-GAPDH_R** | CTCCATGGTGGTGAAGACGC |
| **h-cGas_F** | GGGAGCCCTGCTGTAACACTTCTTAT |
| **h-cGas_R** | CCTTTGCATGCTTGGGTACAAGGT |

**Table S2. List of antibodies used**

| **Antibody** | **Source** | **Identifier** |
| --- | --- | --- |
| **cGAS (mouse)** | CST | 31659S |
| **cGAS (human)** | CST | 15102 |
| **STING** | CST | 13647S |
| **Aβ (MOAB)** | Millipore | MABN254 |
| **PU.1** | CST | 2258S |
| **IBA1 (Rabbit sourced)** | FUJIFILM Wako | 019-19741 |
| **IBA1 (Goat sourced)** | Novus Biologicals | NB100-1028 |
| **LAMP1** | Thermo Fisher | 14-0112-82 |
| **PSD95** | CST | 3450T |
| **Synaptophysin** | CST | 36406T |
| **CD68** | CST | 97778S |
| **Lilrb4** | Invitrogen | PA5-87295 |
| **AXL** | R&D systems | AF854 |
| **PTN** | proteintech | 27117-1-AP |
| **PYCARD** | AdipoGen | AG-25B-0006-C100 |
| **Cleaved-Caspase1** | CST | 4199T |
| **CD11b-APC** | eBioscience | 17-0112-83 |
| **CD45-FITC** | eBioscience | 11-0451-85 |
| **NF-H** | Thermo Fisher | PA1-10002 |
| **β-actin** | CST | 4970S |
